# Substrate specificity and action mechanism of the HerA-NurA nuclease from the hyperthermophilic archaeon *Thermococcus kodakarensis*

**DOI:** 10.1128/mbio.03523-25

**Published:** 2026-02-05

**Authors:** Keishiro Uda, Takeshi Yamagami, Sonoko Ishino, Christoph Gerle, Chai C. Gopalasingam, Hideki Shigematsu, Tomoyuki Numata, Yoshizumi Ishino

**Affiliations:** 1 Department of Bioscience and Biotechnology, Graduate School of Bioresource and Bioenvironmental Sciences, Kyushu University 12923, Fukuoka, Japan; 2 Nagahama Institute of Bio-Science and Technology 53362, Shiga, Japan; 3 Life Science Research Infrastructure Group, RIKEN SPring-8 Center, Hyogo, Japan; 4 Diffraction and Scattering Division, Japan Synchrotron Radiation Research Institute, SPring-8 133704, Hyogo, Japan; 5 Institute of Innovative Research, Institute of Science Tokyo 530262 , Kanagawa, Japan; University of California, Berkeley, Berkeley, California, USA

**Keywords:** archaea, DNA repair, end resection, EM structure, nuclease, translocase

## Abstract

The HerA-NurA complex reportedly functions in DNA end resection in archaea. End resection is important to start homologous recombination by forming a single-stranded DNA region with an overhanging 3′-end, which invades double-stranded DNA (dsDNA) with a homologous sequence to form a D-loop. Here, we studied the structure and functions of HerA-NurA from the hyperthermophilic archaeon, *Thermococcus kodakarensis*. Our analyses demonstrated that NurA is a non-directional and single-stranded specific nuclease, but the HerA-NurA complex cleaves both strands of dsDNA in an exonucleolytic manner, regardless of the structure of the DNA end. The 3D structures of HerA-NurA and its complex with dsDNA revealed the detailed molecular mechanisms of these nuclease reactions. These results suggest that HerA-NurA may not be involved in the end resection process but instead performs other functions, such as exerting an antiviral function by degrading the dsDNA of foreign viruses, similar to recent studies in bacteria.

**IMPORTANCE:**

To understand the specific function of the HerA-NurA complex, which is believed to function in the end resection process to create a 3′-overhanging DNA for the following strand invasion in homologous recombination, we performed biochemical and structural analyses of this complex from a hyperthermophilic archaeon, *Thermococcus kodakarensis*, inhabiting a harsh environment where DNA is easily damaged. We found that the HerA-NurA complex cleaves both strands of double-stranded DNA in an exonucleolytic manner, regardless of the structure of the DNA end. Our structural analysis revealed the detailed characteristics of the nuclease activity exhibited by the HerA-NurA complex. Based on the presented information, it is unlikely that the HerA-NurA complex directly functions in end resection, but rather is involved in other functions, possibly in defense against viral infections.

## INTRODUCTION

Homologous recombination (HR) promotes the exchange of genetic information between chromosomes during meiosis, generating genetic diversity (1). It also plays a vital role in maintaining genome stability as an important DNA repair function. DNA double-strand breaks (DSBs) occur as a result of exposure to exogenous factors such as ionizing radiation or certain chemotherapy drugs, high temperature, or stalled DNA replication, throughout all living organisms. Failure to repair DSBs leads to mutations and chromosomal rearrangements, resulting in the loss of genetic information (2, 3). HR is more accurate than the alternative pathway, non-homologous end joining (NHEJ), because it uses homologous DNA sequences (usually identical sister chromatids) as repair templates and is thought to perform an important function in all three domains of life.

DNA end resection plays a key role in the selection of the NHEJ and HR repair pathways. In Eukarya, the Mre11-Rad50-Nbs1 (human) or Mre11-Rad50-Xrs2 (budding yeast) complex initiates 5′ strand resection, which is further processed by the Dna2 helicase/nuclease and the Sgs1 helicase or Exo1 nuclease (4–7). In Bacteria, the helicase-nuclease complex RecBCD/AddAB initiates HR repair by unwinding and degrading both ends of the DNA. The 3′−5′ exonuclease activity of RecBCD/AddAB is inhibited in the presence of χ sequences, resulting in specific degradation in the 5′−3′ direction and generating a 3′-overhanging single-stranded DNA (ssDNA) (8, 9). However, the *Deinococcus-Thermus* phylum lacks the RecBCD/AddAB system and utilizes the efficient RecFOR system. This repair system primarily uses the 5′-terminal ssDNA exonuclease RecJ in combination with specific helicases, such as RecQ, to efficiently process the broken DNA ends (10, 11), leading to the RecFOR system or the extended synthesis-dependent strand annealing process prior to DNA recombination (12–14).

In Archaea, the Mre11-Rad50 complex reportedly recognizes DSBs, and it is proposed that Mre11 cleaves one strand (5′-strand) at an internal site by its endonuclease activity first, and its 3′–5′ exonuclease starts cleaving from the nick site to the 5′-end. This cleavage process is advantageous if some adduct, such as proteins or chemicals, is bound at the terminus by DSB. Based on this function, the Mre11-Rad50 complex may generate 3′ overhangs at the DNA terminus by itself, but it has been proposed that HerA and NurA, encoded in the same operon with the *mre11* and *rad50* genes in archaeal genomes, act cooperatively to resect the 5′-strand (15–19). The 5′ to 3′ nuclease NurA and the bidirectional DNA helicase HerA have been considered to work for further cleavage of the 5′-strand, instead of the ExoI nuclease and the Dna2-Sgs1 helicase in Eukarya. Structural studies of HerA from *Saccharolobus solfataricus* (SsoHerA) revealed a hexameric ring with a central hole suitable for accommodating double-stranded DNA (dsDNA) (20). However, a heptameric form, which provides a wide channel sufficient for accommodating dsDNA, has also been detected and proposed to function as a DNA loading intermediate (21). Meanwhile, NurA reportedly exhibits Mn^2+^-dependent 5′−3′ exonuclease/endonuclease activity toward both ssDNA and dsDNA (15, 18, 22). The crystal structure of dAMP-Mn^2+^-bound NurA from *Pyrococcus furiosus* (PfuNurA) provided insight into the 5′−3′ orientation of the NurA nuclease (22). Low-resolution cryo-electron microscopy (cryo-EM) data of the SsoHerA-NurA complex indicated that HerA and NurA form a complex at a molar ratio of 6:2, primarily through hydrophobic interactions (20, 23), suggesting that HerA contributes to DNA translocation into the NurA nuclease site. However, although the narrow channel formed between NurA dimers is predicted to accommodate dissociated ssDNA, the structural features required for dissociating dsDNA have not been identified. In other words, the mechanism of the interdependency between NurA and HerA for the nuclease activity remains unclear. Accordingly, the regulatory mechanisms by which the HerA-NurA complex preferentially digests the 5′-end of the DNA duplex and cooperatively provides 3′ ssDNA ends suitable for strand invasion remain poorly understood.

The *herA-nurA* operon is also present in the *Deinococcus-Thermus* phylum in the bacterial domain, likely due to horizontal transfer from Archaea to Bacteria (24). Because *D. radiodurans* lacks Mre11-Rad50, HerA-NurA (DraHN) is predicted to be the minimal end-resection machinery capable of efficiently processing DSB ends. Indeed, cells lacking DraHN exhibit reduced intermolecular recombination efficiency, suggesting its role in HR (24). The physical interaction between DraHerA and DraNurA reportedly results in nuclease activity, whereas DraNurA alone exhibits little nuclease activity (24). Recently, the high-resolution structural analysis of DraHN was reported, and the detailed mechanism of DNA end resection was discussed (25, 26). Compared to the archaeal NurA structure, DraNurA has a unique extended N-terminal region that is involved not only in DraNurA dimerization but also in regulating the helicase and nuclease activities of DraHN. This structure provided new insight into the mechanism of bacterial HN-mediated DNA end resection.

In this study, we performed biochemical and structural analyses of HerA and NurA from the hyperthermophilic archaeon *Thermococcus kodakarensis*. Based on our results, it is difficult to explain how this archaeal HerA-NurA (HN) complex is involved in the 3′-end resection process. Our results suggest that the HN complex may have other functions in HR or functions in other processes; for example, to destroy foreign dsDNA that has entered the cells, rather than the end resection process.

## RESULTS

### HN complex cleaves both strands of dsDNA

**Fig 1:**
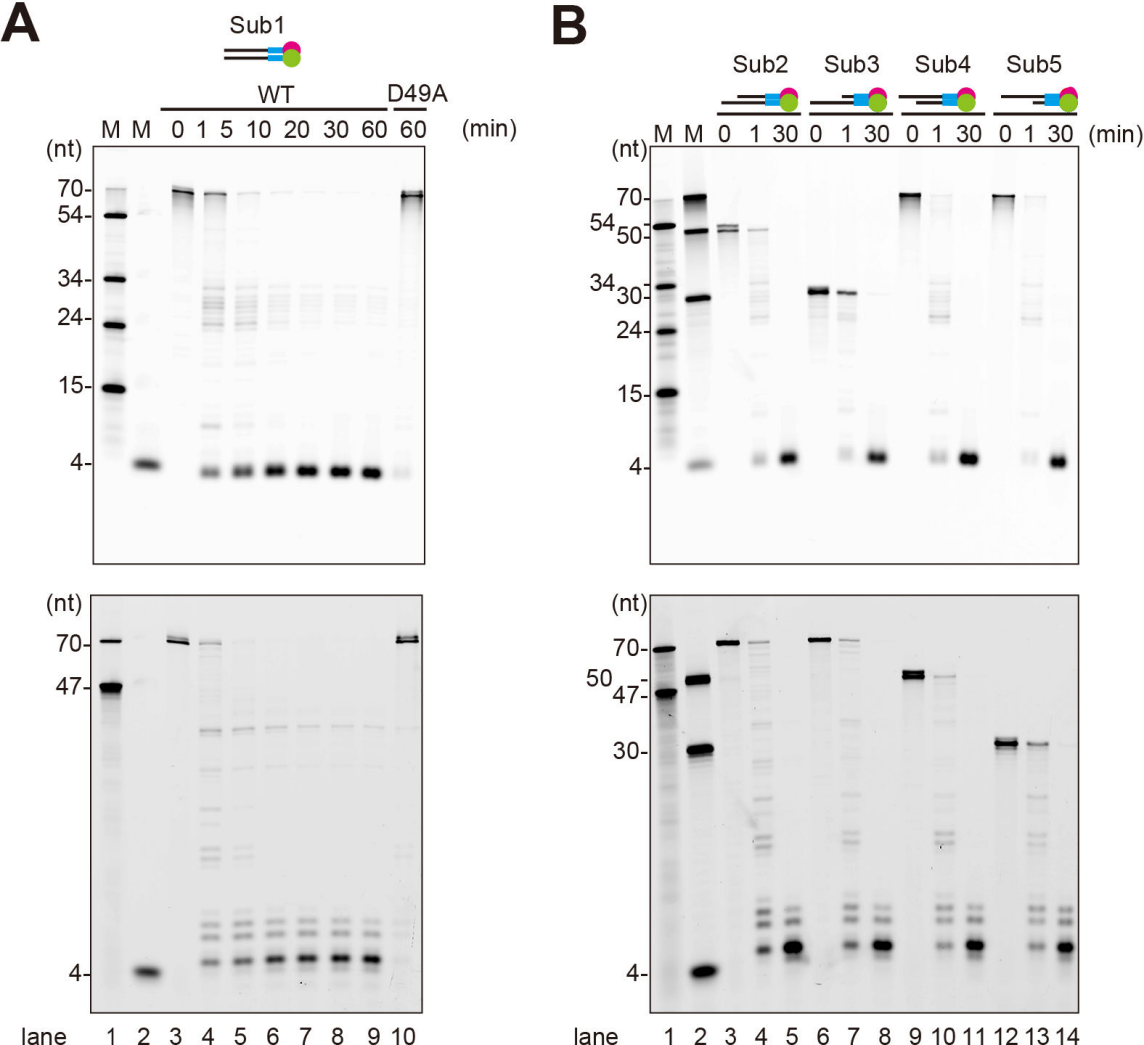
Nuclease activity of the HN complex in the presence of ATP. (**A** and **B**) Time-course analysis of DNA cleavage by the HN complex. D49A refers to the NurA^D49A^-containing HN complex. The substrates are schematically illustrated in each panel. The magenta circle, green circle, and cyan bar represent the 3′-Cy5 labeling, 5′-FAM labeling, and phosphorothioate modification, respectively. More detailed information about the substrates is provided in Fig. S1. The Cy5-detected gel images (top panels) and the FAM-detected gel images (bottom panels) are shown. The reaction time is indicated as a number above each lane. DNA size markers were run in lanes 1 and 2 (indicated as “M”), and their sizes (nt) are shown on the left of the panels.

To investigate the physiological function of the HN complex precisely, we first performed *in vitro* analyses of the nuclease activities of purified NurA and the HN complex from *T. kodakarensis*. Substrate DNAs harboring four phosphorothioate linkages in the terminal regions were labeled with FAM and Cy5 at the 5′ and 3′ ends, respectively, as shown in Fig. S1. The nuclease assay revealed that the HN complex cleaved both strands of dsDNA and did not seem to generate overhangs (Fig. 1A). To monitor the effect of exogenous contamination contributing to DNA cleavage, the HerA-NurA^D49A^ complex, where one of the predicted active site residues, Asp49, in NurA was substituted with alanine, was used for the same nuclease assays. No nuclease activity was observed with the mutant HN^D49A^ complex, suggesting that the detected DNA cleavage was solely derived from the HN complex. This nuclease activity was metal ion dependent, with Mn²^+^ strongly preferred as compared with other divalent metal ions (Fig. S2). In terms of the DNA structure, nuclease reactions were compared using four dsDNAs with 20-nt or 40-nt overhangs at either the 5′ or 3′ end (Fig. 1B). The cleavage patterns were the same as those observed with the blunt-ended 70 bp dsDNA (Fig. 1A). All the reactions stopped with four nucleotides left from both the 3′ and 5′ ends. These results indicate that the HN complex cleaves the phosphodiester bonds in both strands, regardless of the structure of the DNA end. Considering the way that the substrate DNA contacts with NurA nuclease in the HN complex structure, as shown in the subsequent section, this cleavage reaction is processed by the non-directional exonuclease.

### Basic characteristics of NurA nuclease

The nuclease activity of the HN complex is derived from the NurA protein, and therefore, we analyzed the basic properties of the purified NurA. NurA exhibited ssDNA cleavage activity but did not cleave dsDNA under the same conditions (Fig. S3A). The cleavage of the ssDNA stopped at four nucleotides from the 3′ and 5′ ends, as observed for the dsDNA cleavage by the HN complex in Fig. 1. Even with a 60-fold excess amount, NurA alone required 150 min to complete the cleavage reaction, whereas the reaction by the HN complex completed in only 5 min (compare Fig. 1; Fig. S3A). This result suggests that efficient DNA cleavage is dependent on complex formation and coordination with HerA. An assay using circular DNA substrates, that is, lacking any DNA termini, showed that NurA has endonuclease activity for ssDNA, but not for dsDNA (Fig. S3B and C), as previously reported for NurAs from other archaea (18). Thus, NurA is a non-directional nuclease with strict specificity for ssDNA.

### Effect of HerA on the NurA nuclease activity

**Fig 2:**
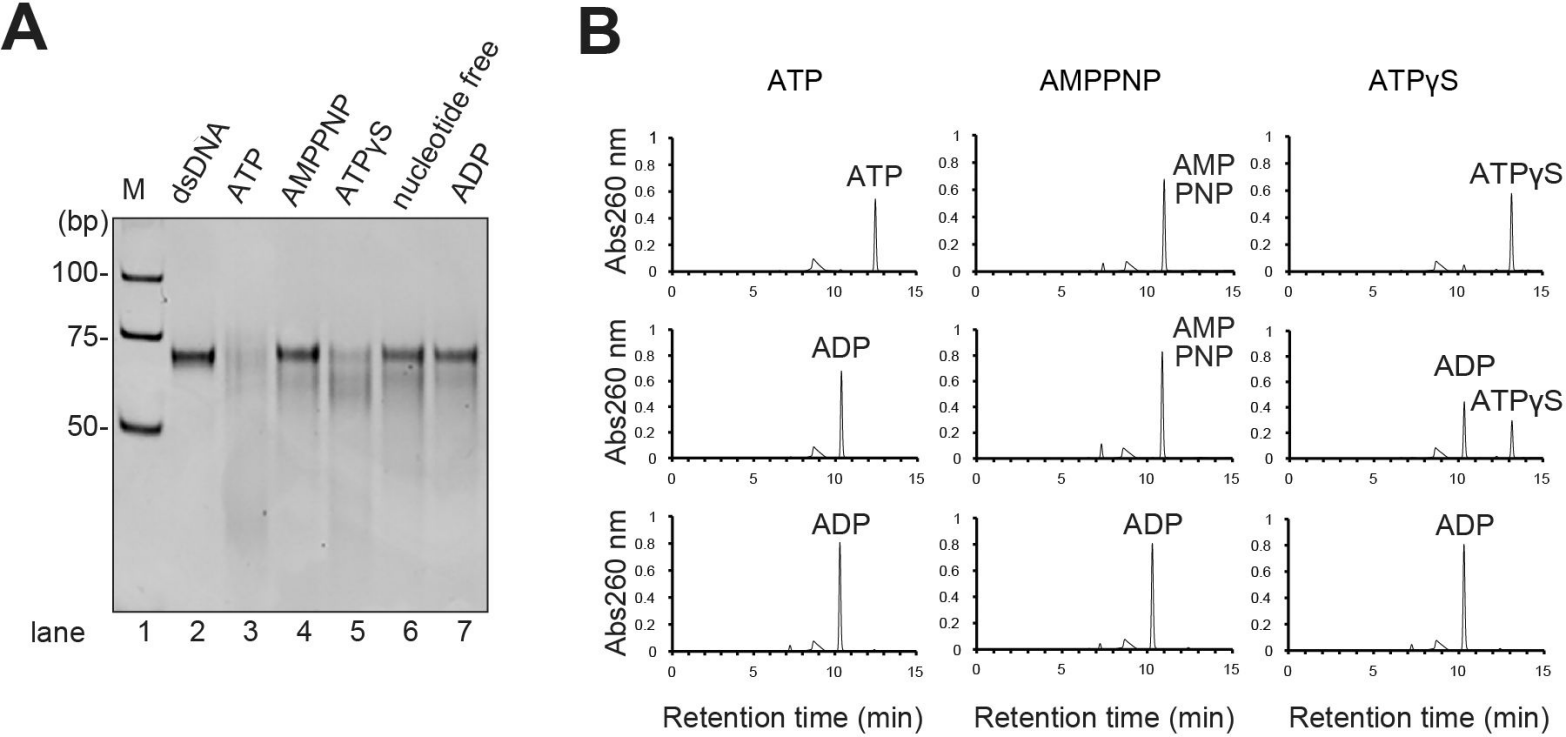
Nucleotide dependency of the nuclease activity from the HN complex. (**A**) The dsDNA cleavage activity of the HN complex in the presence of various nucleotides. The nucleotides used in each reaction are indicated at the top of the gel. M, DNA size markers; dsDNA, substrate as a running control. (**B**) Nucleotide hydrolysis activities of the HN complex with ATP, AMPPNP, and ATPγS. Hydrolysis was evaluated by high-performance liquid chromatography analysis. Each panel shows a chromatogram with absorbance detection at 260 nm. The top panels display the chromatograms of ATP, AMPPNP, and ATPγS. The middle panels show chromatograms of the reactions with the HN complex at 60°C for 10 min, and the bottom panels present the chromatograms of the reaction product control, ADP.

The dsDNA cleavage reaction by the HN complex in Fig. 1 included ATP to activate the helicase function of HerA. Therefore, we employed the nuclease assay to compare the activities in the presence and absence of ATP. Cleavage products from the dsDNA were detected only in the presence of ATP, in contrast to the case of ssDNA, for which the reaction proceeded regardless of the presence or absence of ATP (Fig. S4). In substrates with overhanging ends, only the protruding single-stranded portion was cleaved in the absence of ATP (Fig. S5), in contrast to the reactions with ATP, which processed the cleavage into the dsDNA regions (Fig. 1B). To determine whether ATP hydrolysis is required for the dsDNA cleavage, reactions with various ATP analogs were performed. Cleavage of dsDNA was not observed in the presence of AMPPNP, ADP, or in the absence of ATP (Fig. 2A). In the case of ATPγS, the cleavage reaction proceeded slowly, consistent with the HN complex hydrolyzing ATPγS less efficiently than ATP (Fig. 2B). Thus, it became clear that energy from ATP hydrolysis is required for the HN complex to cleave dsDNA. In summary, the HN complex exhibited non-directional nuclease activity for ssDNA, without requiring ATP. However, it was much more processive than NurA alone (see Fig. 1; Fig. S3). For dsDNA, the ATPase activity of HerA is necessary, presumably to unwind dsDNA and deliver the strand end to the active site of NurA.

### Structures of the HN complex with AMPPNP

**Fig 3:**
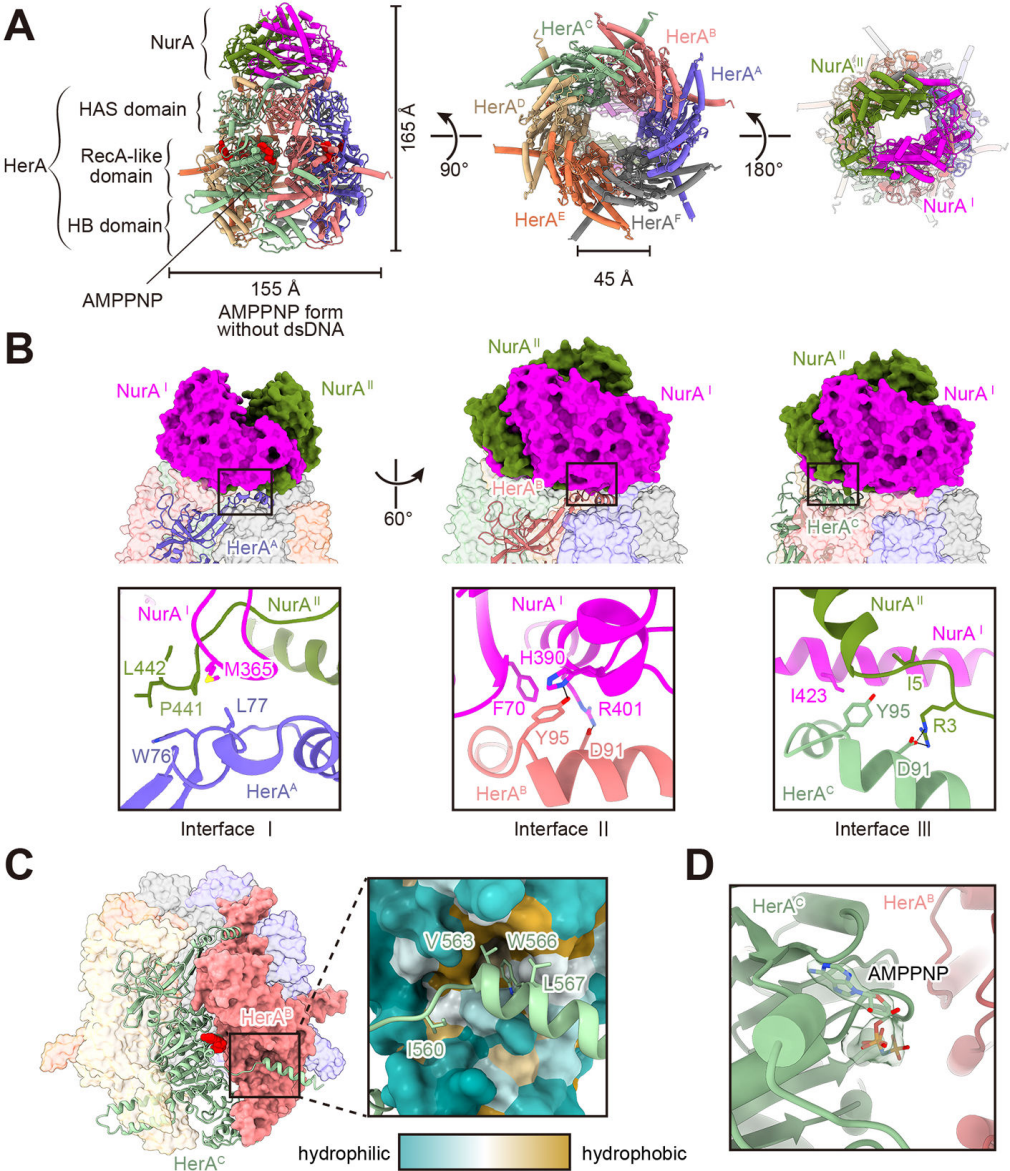
Cryo-EM structure of the HN-AMPPPNP complex. (**A**) Overall structure of the complex in the AMPPNP form without dsDNA, comprising two NurA subunits and six HerA subunits. Bound AMPPNP molecules are colored red (left). The bottom view (middle) and the top view (right) are shown. (**B**) The interaction between NurA and HerA. (Top) Two NurA subunits and five of six HerA molecules are shown in a surface model, and the HerA^A^ (left), HerA^B^ (middle), and HerA^C^ (right) subunits are represented in cartoon models. (Bottom) Close-up view of the interfaces. Key residues are shown as stick models. Hydrogen bonds and salt bridges are indicated by dotted lines. (**C**) The interaction between HerA protomers. Interacting HerA protomers are shown as a green cartoon and a dusty rose surface model. AMPPNP molecules are represented as red spheres. The right panel presents a close-up view of the interaction interface, with the surface colored according to hydrophobicity, based on the Kyte-Doolittle scale, to visualize the hydrophobic interaction pocket. Key interacting residues are shown as stick models. (**D**) The bound AMPPNP molecule is shown, with the corresponding cryo-EM density map displayed.

While crystal structures of archaeal HerA and NurA have been determined individually, and a low-resolution (7.4 Å) cryo-EM structure of the complex has been reported (23), high-resolution structures of the HN complex have remained elusive. To elucidate the mechanisms of complex assembly and the cleavage reaction on dsDNA, the purified HN complex with AMPPNP was subjected to cryo-EM single-particle analysis, and a 3D structure was determined at 2.81 Å resolution (Fig. S6). The HN complex is a hetero-octamer consisting of two NurAs and six HerAs, forming a bullet-shaped structure of approximately 155 Å in width and 165 Å in height and exhibiting *C*_2_ symmetry (Fig. 3A). The bottom region of the HerA hexamer forms an approximately 45 Å diameter pore, and the central tunnel passes through the hexamer ring to reach the NurA dimer. Each HerA protomer comprises three structural domains: the HAS (HerA-ATP Synthase) domain, the RecA-like domain, and the HB (four-helix bundle) domain (Fig. 3A; Fig. S7A). A three-dimensional variability analysis performed on the particles used for refinement, as implemented in CryoSPARC (27, 28), revealed significant flexibility in the HB domain (Movie S1), which is inferred to be involved in DNA binding from the crystal structure of SsoHerA hexamer reported previously (20). The NurA dimer, which adopts a pyramidal architecture, interacts with the top face of the HerA hexamer (Fig. 3B). Each HerA protomer is stabilized through interactions mediated by the C-terminal brace of the adjacent HerA protomer, which predominantly involves hydrophobic contacts (Fig. 3C), reinforcing inter-protomer stability. Furthermore, the cryo-EM maps clearly revealed six AMPPNPs, with one residing in the RecA-like domain of each HerA protomer, which were all located at the interfaces between HerA protomers (Fig. 3A and D).

### Complex interaction between HerA and NurA

The HN complex exhibits *C*_2_ symmetry, in which each set of three HerA subunits (A–C and D–F) interacts with both NurA subunits (I and II) through three distinct interfaces (Fig. 3B). The HAS domain of HerA plays an important role in the NurA interactions, involving all α1 helices and their preceding loop regions. The first interface is predominantly stabilized by hydrophobic interactions between Trp76 and Leu77 of the A subunit (denoted as HerA^A^) and Met365 of the I subunit (denoted as NurA^I^), as well as Pro441 and Leu442 of NurA^II^. The second interface comprises hydrophobic interactions between Tyr95 of HerA^B^ and Phe70 of NurA^I^. Tyr95 of HerA^B^ forms a hydrogen bond with His390 of NurA^I^. Furthermore, a salt bridge is observed between Asp91 of HerA^B^ and Arg401 of NurA^I^. In the third interface, Tyr95 of HerA^C^ forms hydrophobic interactions with Ile423 of NurA^I^ and Ile5 of NurA^II^. This interface is further stabilized by a salt bridge between Asp91 of HerA^C^ and Arg3 of NurA^II^. In the bacterial homologs, hydrophilic interactions are reportedly crucial for the assembly of HerA and NurA (25, 26). In contrast, a previous report posited that the formation of the archaeal HN complex relies primarily on hydrophobic interactions (29). All the reported hydrophobic and hydrophilic residues important for the interactions are conserved in *T. kodakarensis* (Fig. S7B) and contribute to complex stabilization (Fig. 3B). These findings suggest that the interaction mode between NurA and HerA is not uniform among archaea and may exhibit greater diversity than previously anticipated.

### Structural rearrangement of HB domain upon dsDNA binding

**Fig 4:**
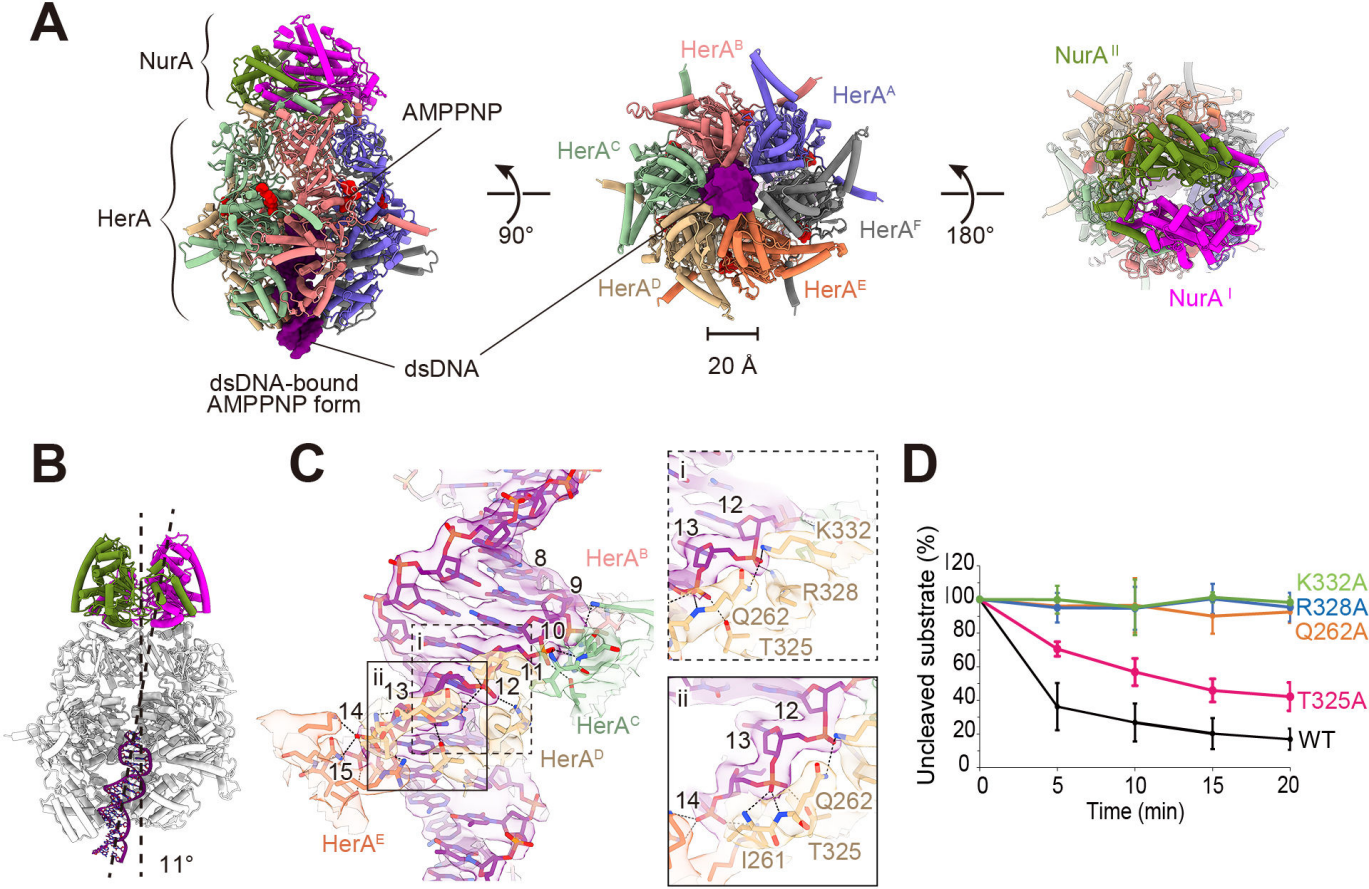
Cryo-EM structure of the HN-AMPPPNP complex with dsDNA. (**A**) Overall structure of the dsDNA-bound HN complex, AMPPNP form. dsDNA observed within the central tunnel formed by the HerA hexamer is indicated as a surface model colored purple (left). The bottom view (middle) and the top view (right) are shown. (**B**) Structural features of the dsDNA bound to HerA. For clarity, two subunits of HerA were omitted, and the remaining are shown in light gray. The dsDNA in the complex is tilted by approximately 11° with respect to the vertical axis of the HN complex. (**C**) Interactions between HerA and dsDNA. (Left) Residues from four HerA subunits interacting with dsDNA are shown. The numbers indicate the nucleotide positions from the end of the DNA bound to HerA. (Right) Close-up view of a representative interaction between HerAD and dsDNA, focusing on the 12th nucleotide (i) and the 13th nucleotide (ii). (**D**) Nuclease activities of HN complexes with HerA mutants. The assay was performed under the same conditions as in Fig. 1. The intensity of the remaining uncleaved substrate was measured as a percentage of the total intensity in each lane on the gel. Each experiment was conducted independently in triplicate, and the error bars represent the standard error of the mean.

Since dsDNA cleavage occurs in the presence of ATP, the HN complex was incubated with a 70 bp dsDNA under conditions containing AMPPNP, and the resulting complex was subjected to structural analysis. The determined structure at 2.30 Å resolution (Fig. S8; Table S1 and S2) revealed that six AMPPNPs are bound to the HerA RecA-like domains (Fig. 4A). The bottom of the HerA hexamer ring serves as an entrance for capturing the dsDNA, and a cryo-EM map corresponding to the dsDNA extends to the middle region of the RecA-like domain (Fig. 4). This observation suggests that approximately 20 bp of the dsDNA entered the central tunnel of the HerA ring (Fig. 4B). dsDNA binding induces a conformational rearrangement of the HerA hexamer, thereby disrupting the symmetrical architecture observed in the form without DNA. The entrance of the HerA tunnel narrows to approximately 20 Å in diameter, and the subunits adopt a spiral staircase-like configuration to accommodate the dsDNA (Fig. 4A). The conformational differences of each HerA protomer with or without dsDNA are localized in the HB domains (Fig. S9). The HB domain structures from the corresponding subunits across the complexes superimpose well, with root-mean-square deviations of 0.54–0.71 Å (100 aligned Cα atoms). This finding suggests that a rigid-body structural shift of the HB domain relative to the remaining regions in HerA may facilitate the snug accommodation of the dsDNA. This domain-specific plasticity is likely enabled by a flexible linker connecting the RecA-like and HB domains. Since the HerA hexamer adopts an asymmetric configuration, the bound dsDNA tilts by approximately 11° from the vertical axis (Fig. 4B). This tilt results in asymmetric interactions with the HerA subunits by which the dsDNA makes close contacts with four out of six HerA molecules (Fig. 4C). The interactions occur through hydrogen bonds with the phosphate backbone, reflecting the non-specific action of HerA on DNA sequences. The binding modes of these four protomers are similar, with each contacting two consecutive phosphate groups (Fig. 4C). In this arrangement, the side chains of Gln262, Arg328, and Lys332 interact with one phosphate group, while that of Thr325 engages the following phosphate group. The nitrogen atoms of the main chain of Ile261 and Gln262 may strengthen the interactions by forming hydrogen bonds with the nearby phosphate groups. The individual Q262A, R328A, and K332A mutations markedly impaired the dsDNA cleavage activity (Fig. 4D), while the T325A mutation moderately reduced it. Accordingly, these residues are directly implicated in actual contributions to the interactions with dsDNA. All six AMPPNPs in HerA remained intact in the structure. Considering the biochemical results described above and our structural findings, the structure of the AMPPNP-bound form likely represents a pre-translocation state, in which the dsDNA is stalled in the mid-region of the HerA tunnel. Further translocation and subsequent cleavage of the DNA appear to require energy derived from ATP hydrolysis.

### Translocation mode of the HN complex along dsDNA

**Fig 5:**
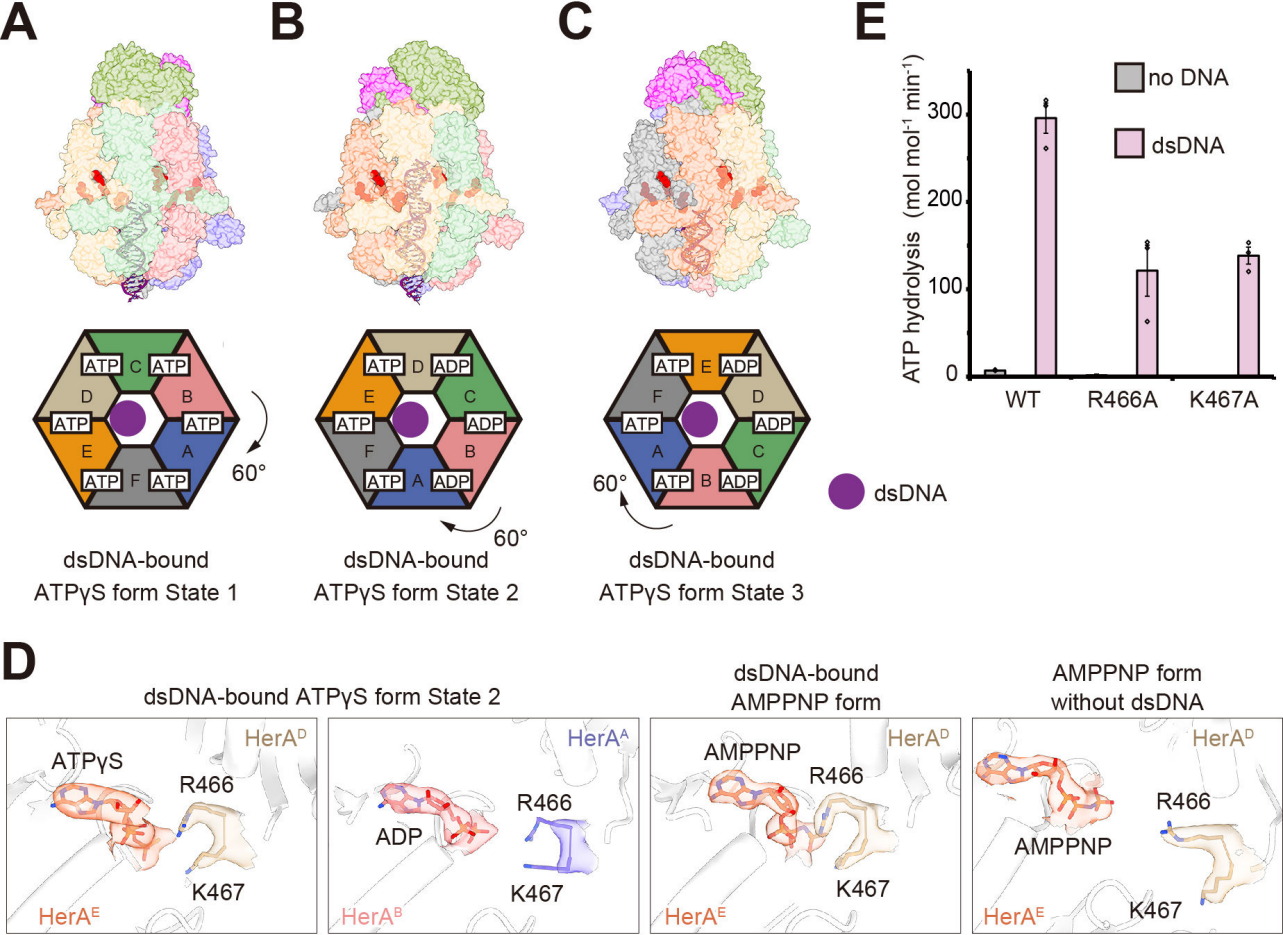
Cryo-EM structures of the HN-ATPγS-dsDNA complex. (A–C) The dsDNA-bound complex in three distinct states (states 1–3) was derived from a single data set. (Top) Surface representations of the HN complex. The dsDNA is shown in a cartoon model. The bound nucleotides are represented as red spheres. (Bottom) Schematic representations of the HerA hexamer, viewed from the bottom of the complex. The subunit arrangement, the bound nucleotides, and the dsDNA are indicated. The color codes are consistent with those in Fig. 3. (**D**) Close-up views around the ATP-binding site between chains D and E (leftmost), and chains A and B (second to left) in dsDNA-bound ATPγS form state 2, chains D and E in dsDNA-bound AMPPNP form (second to right), and chains D and E in AMPPNP form without dsDNA (rightmost). The R466 and K467 residues, which are from adjacent subunits, are represented as stick models. The cryo-EM density maps corresponding to these two residues and ATP analogs are overlaid and colored according to each model. (**E**) Effects of HerA mutations on the ATPase activity of the HN^D49A^ complex. ATP hydrolysis was measured in the presence or absence of dsDNA. The HerA mutations in the HN^D49A^ complex are shown below the bars. Three independent experiments were conducted. The error bars represent the standard error of the mean, and the individual data points are plotted.

The HN complex hydrolyzes ATPγS slowly and shows weak cleavage activity for dsDNA in the presence of this ATP analog (Fig. 2). To elucidate the structural dynamics of the HN complex during the translocation process, we conducted a structural analysis using ATPγS and a hairpin-forming dsDNA substrate, whose loop structure at one end physically blocks the entry into the central tunnel of HerA. Three distinct structures (states 1, 2, and 3) containing dsDNA, ATPγS, and its hydrolyzed product were obtained at 3.09 Å, 2.97 Å, and 3.14 Å resolutions, respectively (Fig. S10 and S11). Their cryo-EM maps correspond to dsDNA in the HerA tunnel fitted to 20 bp for State 1, 30 bp for State 2, and 13 bp for State 3. Intriguingly, superimposition of the dsDNAs in each structure revealed a rotational shift of the NurA dimer by about 60° across the three observed states, although the interactions between the NurA dimer and the HerA hexamer remained stable, indicating that the complex translocates as a single unit (Fig. 5). Similar to the structure of the AMPPNP form, the dsDNA axis in these three structures is tilted by 11° relative to the vertical axis of the HN complex (Fig. 5A through C). Consequently, the dsDNA does not engage with all six HerA subunits simultaneously but interacts selectively with four HerA subunits. Specifically, the dsDNA contacts subunits C–F in state 1, A and D–F in state 2, and A, B, E, and F in state 3 (Fig. 5A through C). This sequential shift in interacting subunits is consistent with the incremental 60° rotation of the HN complex during the translocation process. All six nucleotide-binding sites were occupied by ATPγS in State 1, but three ATPγSs and three ADPs were found in states 2 and 3. The ATPγSs were located at the interfaces between subunits A and F, D and E, and E and F in state 2, while they were detected between subunits A and B, A and F, and E and F in state 3 (Fig. 5A through C; Fig. S12). This observation indicates that the reaction turnover of ATP hydrolysis is remarkably fast, although the order in each subunit is not known. These findings suggest a mechanism in which the DNA strand in the HerA tunnel is transported to the nucleolytic active site of NurA by the 60° stepwise rotational movement, using ATP hydrolysis as the driving force.

### Basic residues in HerA affecting ATPase activity

In the dsDNA-bound conformation, two basic residues, Arg466 and Lys467, from an adjacent HerA protomer form hydrogen bonds with the β- and γ-phosphates. In contrast, in the dsDNA-unbound conformation, these basic residues are positioned far from the nucleotide, highlighting a pronounced structural divergence (Fig. 5D). In addition, these two residues are far from the ADP, even in the DNA-bound form (Fig. 5D). In the whole structure of the HerA hexamer, these residues and ATPγS are separated in some subunits even in the presence of DNA (Fig. S12). Since DNA successively contacts different HerA subunits, it is not surprising that Arg466 and Lys467 are close to the nucleotide triphosphate in some subunits and farther away in others. Based on this observation, we hypothesized that these two residues may facilitate or sense ATP hydrolysis. We measured the ATP hydrolysis of the HerA mutants complexed with NurA^D49A^. The HN^D49A^ complex (wild-type HerA in the complex) exhibited only weak ATPase activity in the absence of DNA; however, this activity increased approximately 50-fold when dsDNA was added (Fig. 5E). Alanine mutations of the HerA R466 and K467 residues resulted in a marked reduction, indicating their critical role in hydrolysis.

### DNA unwinding by NurA in the complex

**Fig 6:**
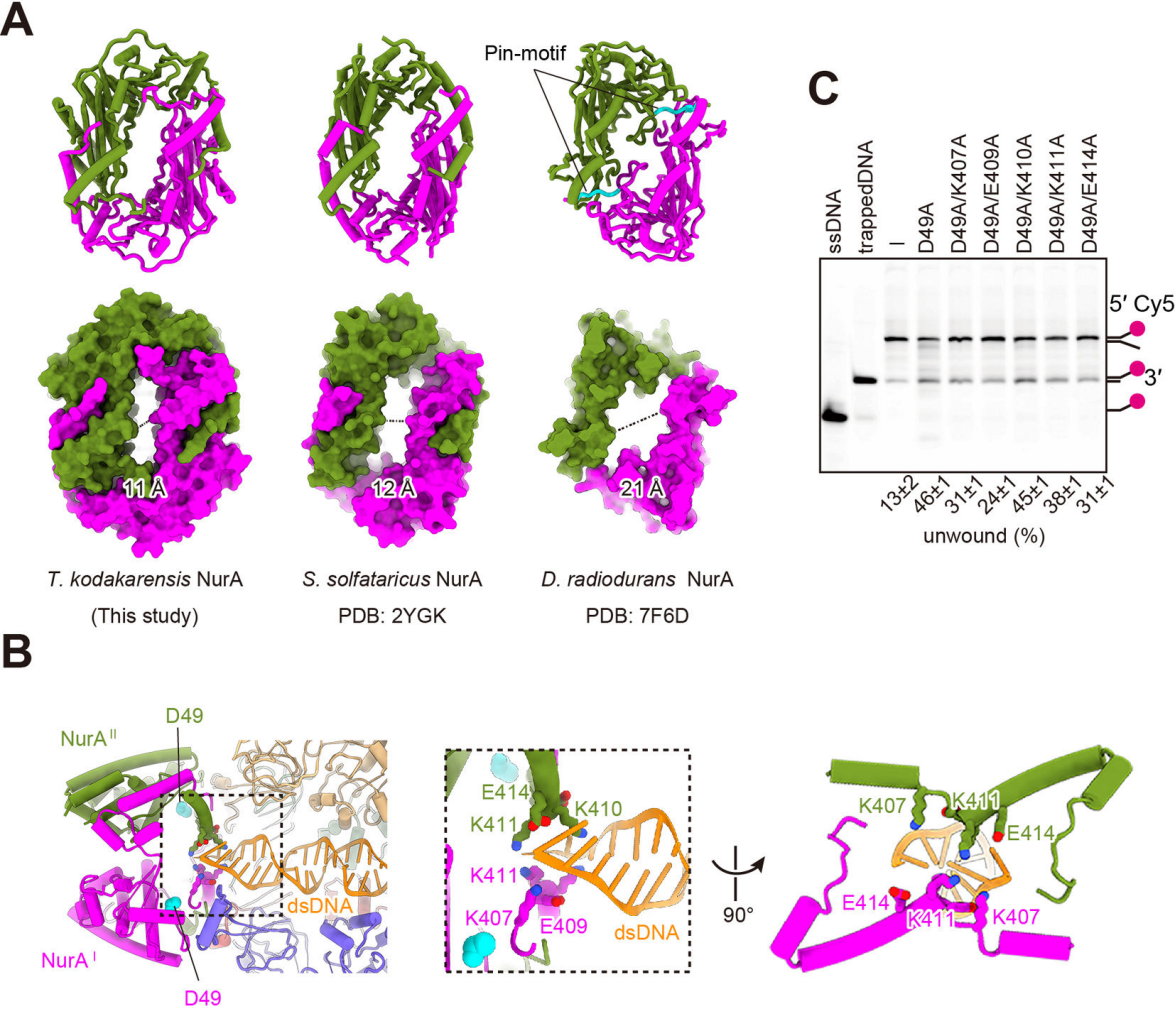
Exploring the role of NurA in DNA duplex unwinding. (**A**) Structural comparison of NurAs from different species. The NurA dimers derived from *T. kodakarensis* (left, this study), *S. solfataricus* (middle, PDB ID: 2YGK), and *D. radiodurans* (right, PDB ID: 7F6D) are shown. All structures are viewed from the bottom side of the NurA dimer. The narrowest region of the tunnel within the dimer is indicated by dotted lines. The pin motif observed in DraNurA, which is crucial for dsDNA unwinding, is highlighted in cyan. (**B**) (Left) Structural model of the dsDNA-HN complex with dsDNA approaching the NurA entry site, derived from the dsDNA-bound ATPγS form state 2 structure. For clarity, the two HerA subunits were omitted, and the DNA is colored orange. The Asp49 residue of NurA, an essential residue for catalytic activity, is shown as a cyan sphere. (Middle) Close-up view of the left panel. (Right) Close-up view of the middle panel, rotated by 90°. Potential steric clashes between the dsDNA and the entry site of the NurA dimer are expected. NurA residues likely to clash with the dsDNA are labeled and shown as stick models. (**C**) Effects of NurA mutations on the helicase activity of the HN complex. The NurA mutations are indicated in each lane. The quantified helicase activities are shown at the bottom as the relative amount of unwound DNA (%) from three independent experiments. Single-stranded and trapped DNAs were loaded in parallel as controls. Incubated substrate without protein is indicated as “−.” The DNA structures corresponding to each band are indicated on the right side. The magenta circle represents the Cy5 labeling at the 5′ terminus.

Models of the reaction mechanism for DNA cleavage, in which the dsDNA dissociation could occur at the entrance, middle, or exit site of the HerA hexamer tunnel, have been proposed (23, 29). As described above, we determined three distinct structures of the dsDNA-HN complex, using ATPγS (Fig. 5A through C). The DNA remained double-stranded in all structures, and no dissociated ssDNA near the exit of the tunnel was observed. Furthermore, no wedge-like structure that could facilitate dsDNA unwinding was observed within the HerA tunnel. Because the NurA nuclease is strictly ssDNA specific, as shown above, the dsDNA should be dissociated into single strands before entering the active site of NurA. The bottom region of the NurA dimer forms an oval-shaped pore, and the narrowest region is approximately 11 Å (Fig. 6A). We constructed a structural model in which the dsDNA was extended to the oval-shaped entrance of the NurA dimer, using the state 2 structure, which provided the clearest DNA density. The model suggested that while the extended DNA did not directly interact with HerA, it appeared to sterically clash with the C-terminal region of NurA. Five candidate residues, Lys407, Glu409, Lys410, Lys411, and Glu414, of NurA are involved in interactions with dsDNA and its dissociation (Fig. 6B). To assess their potential role in DNA unwinding, we prepared alanine substitution mutants of NurA^D49A^ and evaluated the dissociation ability of the HN complex, using a splayed-arm DNA labeled with Cy5. Under conditions where approximately 45% unwound products were obtained by the HN^D49A^ complex, the K407A, E409A, K411A, and E414A mutants each showed clearly reduced activity for strand dissociation (Fig. 6C). These results suggest that NurA in the HN complex is involved not only in cleavage but also in double-strand dissociation.

## DISCUSSION

Although many previous reports related to the HN complex described its function in archaeal end resection, it remained unclear whether the complex indeed plays a direct role in this process (reviewed in references 30–33). To address this important question, we conducted a comprehensive evaluation of the DNA cleavage mode of the HN complex from *T. kodakarensis* and investigated its DNA processing mechanism. Our biochemical and structural analyses yielded several key insights into the functional properties of the complex. First, we confirmed that NurA basically possesses a non-specific nuclease activity strictly for ssDNA. Unexpectedly, the HN complex cleaved both strands of dsDNA non-directionally regardless of the terminal end structure, contrary to the expected generation of the 3′-overhang structure. Previous studies used dsDNA substrates in which only one of the two strands was labeled, raising the possibility that both the labeled and unlabeled strands were cleaved, but only the cleavage products from the labeled strand were detected. A study of the HN complex from *D. radiodurans* employed a DNA strand labeled with two different fluorophores at both termini, which self-annealed to form a hairpin-shaped DNA substrate. This report showed that both strands were cleaved (26), consistent with our results.

In the present study, we determined high-resolution structures of the archaeal HN complex and its dsDNA-bound form for the first time. Consistent with previous studies of the bacterial and archaeal HN complexes, the *T. kodakarensis* HN complex is composed of two NurA subunits and six HerA subunits. In DNA-bound structures, HerA formed a hexameric ring, with the DNA positioned within the central tunnel of the complex. Comparative structural analyses showed that the dsDNA was fed more extensively toward the entrance of the NurA dimer channel in the ATPγS-bound form, as compared with the AMPPNP-bound form. These findings unambiguously demonstrate that ATP hydrolysis is coupled to DNA translocation by the HN complex.

We compared the structures of the HerA hexamer from different archaeal species and found that the diameters of their holes are large enough to allow the dsDNA chain to move all the way for translocation to the entrance of NurA by conformational change using energy from ATP hydrolysis. This feature appears to be common regardless of whether in thermophilic or mesophilic archaea (Fig. S13). Furthermore, we showed that the HB domain of *T. kodakarensis* HerA flexibly moves for substrate DNA capturing. This feature is probably conserved in SsoHerA, judging from the reported crystal structure, in which the *B*-factor is higher in the HB domain as compared with other regions (Fig. S14).

FtsK is an ATP-dependent DNA translocase that contributes to accurate chromosome segregation during cell division in bacteria, and it belongs to the same family as HerA, named the “FtsK-HerA superfamily” (34). The results from a cryo-EM analysis of FtsK bound with dsDNA and various ATP analogs (35) were basically similar to those from our analysis of HerA.

Mutational and biochemical analyses of several residues of NurA, predicted to be involved in DNA interactions, resulted in reduced helicase activity. In particular, alanine substitutions of Lys407, Glu409, and Glu414 led to a marked decrease in activity. A previous study of SsoNurA suggested that it may function like a “plowshare” for DNA unwinding (29). Similarly, deletion of the N-terminal region of DraNurA reportedly abolished unwinding (26). These findings support the premise that NurA is involved in DNA unwinding.

**Fig 7:**
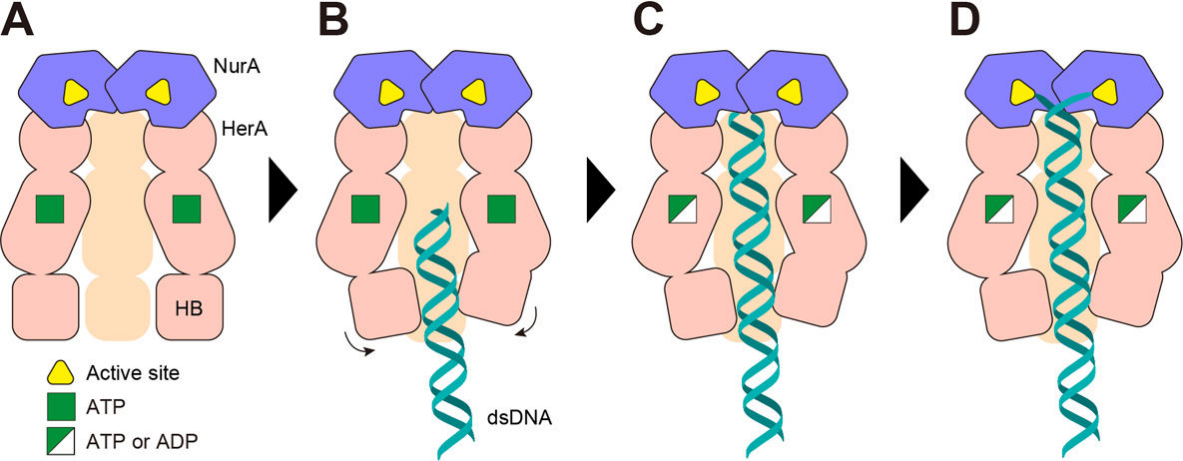
Model for the mechanism of dsDNA cleavage by the archaeal HN complex. NurA, HerA, and the substrate dsDNA are schematically illustrated. (**A**) In the state without DNA, the HN complex exhibits symmetry, and the HB domains at the bottom of the HerA hexamer flexibly move to form a tunnel for capturing DNA. (**B**) Upon dsDNA binding, the HB domains undergo conformational changes to align along the dsDNA and establish interactions suitable for translocation. (**C**) The DNA strand moves through the HerA tunnel, using the energy generated by ATP hydrolysis by HerA. (**D**) The dsDNA is dissociated at the entrance of the NurA dimer, and each ssDNA is delivered to the active center of NurA by successive translocations.

Based on our findings, we propose the following model (Fig. 7). In the absence of DNA, the HN complex maintains a flexible conformation, in which the HB domain of HerA exhibits dynamic motion, presumably to facilitate substrate capture. Upon dsDNA binding, the HB domain undergoes a conformational change with helical rearrangement. Subsequently, ATP hydrolysis drives the stepwise rotational movement of the complex in 60° increments, thereby translocating the DNA toward the active site of NurA. Given that each HerA subunit interacts with two nucleotides of dsDNA, it is presumed that each 60° rotation leads to a 2-nucleotide shift of DNA toward NurA. This observation is consistent with the 12 bp pitch of the HerA-bound DNA strand. The dsDNA is guided into the active sites of the two NurA subunits, where each strand is cleaved sequentially or simultaneously.

Our results indicate that the HN complex is unable to generate the 3′-overhangs required for HR. A previous study of the *Sulfolobus* HN complex proposed that the digestion mode changes depending on the DNA end structure (29). For blunt ends or short overhangs (<5 bases), it acts in a double-strand digestion mode to destroy foreign DNA as a defense system, whereas for longer overhangs, an HR repair pathway operates in a single-stranded digestion mode. This idea derives from the analogy to bacterial RecBCD, which digests both dsDNA and ssDNA, with the χ sequence serving as the switch for functional conversion (8, 36). In the case of the archaea, the generation of a sufficiently large 5′-overhang by the 3′−5′ exonuclease activity of Mre11 may trigger the function of the HN complex to produce 3′-overhang structure (29). Our experimental results demonstrated that double-strand cleavage predominantly occurred, even for substrates with long overhangs (20 mer and 40 mer), and did not fit the hypothesized model. However, it would be interesting to investigate the effect of Mre11 on the reaction of the HN complex.

Since the HN complex cleaves both strands of dsDNA regardless of the DNA structure, it is not trivial to predict the function of this complex in the end resection process from our current study. Genetic studies revealed that Δ*nurA*Δ*herA* strains of *T. thermophilus* did not exhibit increased sensitivity to UV irradiation but instead displayed enhanced resistance (37). In the case of *D. radiodurans*, disruption of *nurAherA* conferred ~10-fold enhanced resistance to a high dose of far-ultraviolet light (600 J/m^2^) and a high concentration of mitomycin C (MMC; 40 μg/mL) (26). However, another report on *D. radiodurans* showed that the *nurA* and *herA* knockouts led to abnormal cell proliferation and reduced intermolecular recombination efficiency but had no obvious effect on radioresistance (24). These results do not necessarily indicate that HerA-NurA plays an essential role in the end resection process. In archaea, the *nurA* and *herA* genes are essential for the viability of *Saccharolobus islandicus* (19, 38). The *nurA* and *herA* genes could not be disrupted in *T. kodakarensis* in our earlier work (39). In *S. islandicus*, HerA has been reported to interact with PINA (ATPase/DNA helicase) and Hjc (Holliday junction resolvase), suggesting that the HN complex may play important roles in HR process other than end resection and in repair of stalled replication forks (19, 40). In other cases, the ssDNA binding protein, single-stranded binding protein (SSB) in *S. tokodaii* (41) and *S. solfataricus* (42), interacts with HerA to regulate the nuclease activity of the HN complex. It has also been reported that the 5′−3′ exonuclease activity of RecJ in *D. radiodurans* is stimulated by interaction with HerA (43). In the case of halophilic archaea, the *nurA* and *herA* are not apparent. The Δ*mre11* and Δ*rad50* strains have been isolated from them. In *Halobacterium* sp. NRC-1, *mre11* and/or *rad50* deletion mutants do not decrease in survival by irradiations of UV-C and γ-ray irradiations or treatment with alkylating agents compared with the wild-type strain. However, the Δ*mre11*, but not Δ*rad50*, strain exhibited a reduced rate of repair from double-strand DNA break, suggesting a Rad50-independent function of Mre11 in DNA DSB repair (44). In addition, *H. volcanii* lacking Mre11-Rad50 is more resistant to DNA damage than wild-type cells, suggesting that Mre11 and Rad50 may function to regulate the progression of HR (45). Therefore, it is also possible that HerA and NurA, which are expressed together with Mre11 and Rad50 from the same operon, may have a similar regulatory function other than end resection. Another point to consider is that, as already mentioned, HerA and FtsK are the same family proteins with similar structures. *D. radiodurans* has both HerA and FtsK, but archaea lack FtsK (46). The archaeal HerA is possible to play an important role in cell division. It is difficult to comprehensively compile these data to understand the functions of the HN complex, but it may have evolved in diverse ways in Archaea and Bacteria.

Recent studies reported that the bacterial HerA interacts and forms complexes with nucleases such as Sir2 (47, 48) and DUF4297 (49), and the coordinated activity of HerA and its associated nuclease is fundamentally important for antiviral responses. The cleavage mode of the *T. kodakarensis* HN complex may reflect a potential role in antiviral defense in archaea, similar to that observed in bacteria.

Upon viral infection, Csa3a, a CRISPR-associated protein, becomes activated and binds to the promoter region of the CRISPR locus, stimulating the transcription of downstream genes and thereby contributing to the elimination of foreign genetic elements (50). A study conducted in *S. solfataricus* demonstrated that Csa3a binds to the promoter regions of the *nurA* and *herA* genes (51), suggesting that the expression of these genes may be upregulated upon viral infection, leading to the degradation of the viral DNA. Additionally, a *nurA-csm6-herA* gene cluster is associated with the CRISPR arrays in several species of the *Pyrobaculum* genus (52), suggesting their involvement in the antiviral defense system. Taken together, these observations suggest that the HN complex also contributes to antiviral defense systems in *T. kodakarensis*. It will be very interesting to determine whether the HN complex plays a defensive role against intruding viruses in *T. kodakarensis* cells.

## MATERIALS AND METHODS

### Recombinant protein preparation

The *nurA* (TK_RS11105) and *herA* (TK_RS11120) genes were amplified by PCR from *T. kodakarensis* genomic DNA, using the primer sets nurA-F/R and herA-F/R (Table S3), respectively. The PCR products were digested with NdeI and NotI and ligated into the corresponding sites of the pET21a (+) and pET24a (+) vectors (Novagen) using T4 DNA ligase to produce pET21a-TkoNurA and pET24a-TkoHerA, respectively. The HN complex was produced by co-expressing the genes for HerA and NurA in *Escherichia coli* BL21-CodonPlus (DE3)-RIL cells (Agilent Technologies). The transformed cells were cultured in luria broth (LB) medium containing 50 mg/L ampicillin, 50 mg/L kanamycin, and 34 mg/L chloramphenicol at 37°C until the OD_600_ reached 1.0. The expression of the *herA* and *nurA* genes was induced by adding isopropyl β-D-thiogalactopyranoside (IPTG) to a final concentration of 1 mM, and the cells were further cultured at 25°C for 18 h. The cell lysate was prepared by sonicating the harvested cells in buffer A (50 mM Tris-HCl, pH 8.0, and 0.1 mM EDTA) containing 0.1 M NaCl. After centrifugation at 23,670 × *g* for 10 min, the supernatant was incubated at 80°C for 20 min, and the heat-resistant fraction was then obtained by centrifugation. Polyethyleneimine was added at a concentration of 0.15% (wt/vol) to precipitate nucleic acids in the heat-resistant fraction. After the supernatant was obtained by centrifugation at 23,670 × *g* for 10 min, ammonium sulfate was added to 80% saturation to precipitate proteins. After centrifugation at 23,670 × *g* for 10 min, the precipitates were resuspended in buffer A containing 1 M (NH_4_)_2_SO_4_ and then applied to a HiTrap Phenyl HP column (Cytiva). Hydrophobic interaction chromatography was performed with a 1–0 M (NH_4_)_2_SO_4_ gradient in buffer A. The HN complex-containing fraction was dialyzed against buffer A containing 0.15 M NaCl and then applied to a HiTrap Heparin HP column (Cytiva), which was developed with a linear gradient of 0.15–1 M NaCl in buffer A. The HN complex-containing fractions were applied to an anion-exchange column (HiTrap Q HP; Cytiva), after dialysis against buffer A containing 0.15 M NaCl. The column was developed with a 0.15–1.0 M NaCl gradient in buffer A. The eluted HN complex was subjected to size-exclusion chromatography on a Superose 6 Increase 10/300 GL column (Cytiva), equilibrated with buffer A containing 0.15 M NaCl. The purified HN complex was concentrated using Amicon Ultra-4 Ultracel-100K centrifugal filters (MERCK). The concentration of the HN complex was calculated by measuring the absorbance at 280 nm with a NanoDrop 2000 and using the extinction coefficient of 569,100 M^−1^ cm^−1^ for the complex in a 2:6 molar ratio. Purified proteins were stored at −80°C. The NurA protein was produced in *E. coli* BL21-CodonPlus (DE3)-RIL cells carrying pET21a-TkoNurA and cultured in LB medium containing 50 mg/L ampicillin and 34 mg/L chloramphenicol. The procedures for the recombinant *E. coli* cell cultivation were the same as those for the HN complex, except the *nurA* gene expression was induced at 0.4 OD_600_. The NurA protein was purified using the same protocol as for the HN complex, involving heat treatment, polyethyleneimine treatment, and ammonium sulfate precipitation, followed by sequential chromatography on HiTrap Phenyl HP and HiTrap Heparin HP columns. The NurA-containing fractions were applied to a cation-exchange column (HiTrap SP HP; Cytiva) after dialysis against buffer A containing 0.15 M NaCl. The column was developed with a 0.15–1.0 M NaCl gradient in buffer A. The purified NurA protein was concentrated using Amicon Ultra-4 Ultracel-30K centrifugal filters. The concentration of the NurA dimer was calculated by the OD_280_ value and an extinction coefficient of 108,420 M^−1^ cm for NurA. The mutations were introduced via PCR-mediated site-directed mutagenesis, using pET21a-TkoNurA and pET24a-TkoHerA as the templates. The primer sets (F and R) used for mutagenesis are shown in Table S3. The designed mutations were confirmed by nucleotide sequencing. The mutant proteins were prepared in the same manner as the wild-type proteins.

### DNA substrate preparation

Oligonucleotides were obtained from Sigma-Aldrich and Eurofins Genomics and are listed in Fig. S1A. The DNA substrates prepared for this study are shown in Fig. S1B. The appropriate oligonucleotides were annealed in a 1:1 combination in 50 mM Tris-HCl, pH 8.0, and 100 mM NaCl. The LoopDNA used for cryo-EM analysis was prepared by self-annealing a long ssDNA.

### Nuclease activity assays

The nuclease activity assays were conducted in mixtures (40 µL) containing 50 mM Tris-HCl, pH 7.0, 100 mM NaCl, 2.5 mM MnCl_2_, 1 mM DTT, 20 nM fluorescent-labeled DNA substrate, and 20 nM HN complex or 1,250 nM NurA (as a dimer), in the presence or absence of 1 mM ATP. The reaction mixtures were incubated at 60℃ for the indicated times, and aliquots (5 µL) were collected. The reactions were stopped by the addition of proteinase K and SDS to final concentrations of 2 mg/mL and 0.1%, respectively. The samples were incubated at 37℃ for 30 min, and after an equal volume of loading solution (98% formaldehyde and 0.1% Orange G) was added, the samples were heated at 98°C for 5 min and then immediately transferred onto ice. The samples were separated by denaturing 15% polyacrylamide gel electrophoresis (PAGE) with 8 M urea in TBE buffer (89 mM Tris, 89 mM boric acid, and 2.5 mM EDTA, pH 8.3). The gel images were visualized by using an Amersham Typhoon scanner (Cytiva). To evaluate the metal ion dependency, a reaction mixture was prepared using 50 mM Tris-HCl, pH 7.0, 100 mM NaCl, 1 mM DTT, 1 mM ATP, 800 nM dsDNA (Sub8), and 800 nM HN complex. Various metal ions were added to a final concentration of 2.5 mM, and the reaction mixtures (10 µL) were incubated at 60°C for 30 min. The reactions were terminated by adding proteinase K and SDS to final concentrations of 2 mg/mL and 0.1%, respectively, followed by an additional 30 min incubation at 37°C. The samples were mixed with 5× loading dye (15% Ficoll, 0.1% xylene cyanol, and 0.1% bromophenol blue) and separated by native 10% PAGE using TBE buffer. The gel was stained with ethidium bromide and visualized under UV illumination. To evaluate the dependency on nucleotide analogs, the reaction mixture (10 µL) was prepared using 50 mM Tris-HCl, pH 7.0, 100 mM NaCl, 2.5 mM MnCl_2_, 1 mM DTT, 800 nM dsDNA (Sub8), and 800 nM HN complex. Each nucleotide analog was added to a final concentration of 1 mM. The reaction and PAGE analysis were performed in the same manner as the metal dependency evaluation. The endonuclease activity assays were conducted in mixtures containing 50 mM Tris-HCl, pH 7.0, 100 mM NaCl, 2.5 mM MnCl_2_, 1 mM DTT, 1 mM ATP, 45 nM phiX174 (ssDNA) or pUC18 (dsDNA), and 2.7  µM NurA protein (as a dimer). The reaction mixtures (10 µL) were incubated at 60℃ for the indicated times. The reactions were stopped by the addition of SDS and proteinase K to final concentrations of 0.1% and 2 mg/mL, respectively. The samples were incubated at 37°C for 30 min, mixed with 5× loading dye, and then separated by 0.8% agarose gel electrophoresis in TAE buffer (40 mM Tris-acetate and 1 mM EDTA, pH 8.3). The gel was stained with ethidium bromide and visualized by UV illumination.

### High-performance liquid chromatography analysis for the hydrolysis of ATP and ATP analogs

The hydrolysis of ATP analogs by the HN complex harboring the D49A mutation in NurA (HN^D49A^ complex) was assessed using high-performance liquid chromatography. The reaction mixture (15 µL) contained 50 mM Tris-HCl, pH 7.0, 100 mM NaCl, 2.5 mM MnCl₂, 1 mM DTT, 1 mM of either ATP, AMPPNP, or ATPγS, 2 µM dsDNA (Sub8), and 1 µM HN^D49A^ complex. Reactions were carried out at 60°C for 10 min and stopped by the addition of EDTA, SDS, and proteinase K to final concentrations of 100 mM, 0.1%, and 2 mg/mL, respectively, followed by an incubation at 37°C for 10 min. An aliquot (10 µL) of the reaction mixture was injected into a YMC-Triart C18 column (4.6 × 150 mm), which was developed with a linear gradient of 7.5%–40% acetonitrile in 50 mM hexylammonium acetate, pH 7.0, for a total volume of 15 mL.

### ATPase activity assay by Pi measurement

The reaction mixture (60 µL) contained 20 mM Tris-HCl, pH 7.0, 100 mM NaCl, 2.5 mM MnCl₂, 1 mM ATP, and 2 µM HN^D49A^ complex, with or without 4 µM dsDNA (Sub8). The reaction mixtures were incubated at 60°C for 0, 5, 10, 20, and 30 min in the absence of DNA, and for 0, 5, 10, 20, 30, and 60 s in the presence of DNA. Aliquots (10 µL) were withdrawn from the reaction and quenched by adding EDTA to a final concentration of 83 mM. The amount of released Pi was quantified using an EnzChek Phosphate Assay Kit (Thermo Fisher Scientific), according to the manufacturer’s instructions. Standard errors of the mean were calculated from three independent experiments.

### Helicase activity assay

The helicase activity was measured in a reaction mixture (10 µL) containing 50 mM Tris-HCl, pH 7.0, 100 mM NaCl, 2.5 mM MnCl_2_, 1 mM DTT, 1 mM ATP, 40 nM DNA substrate (Sub10), 120 nM trap DNA (to prevent re-annealing of the unwound DNA), and 640 nM HN complex. After an incubation at 60°C for 30 min, 5 µL aliquots were taken, and the reactions were terminated by adding SDS and proteinase K to final concentrations of 0.1% and 2 mg/mL, respectively, followed by an incubation at 37°C for 30 min. An aliquot (3 µL) was mixed with 5× loading dye2 (15% Ficoll and 1.25% Orange G). Detection of the activity by native 10% PAGE was performed as described above. The ImageQuant TL software (Cytiva) was used for quantitative analysis. The unwinding efficiency was calculated based on the amounts of substrate and product obtained from three independent experiments.

### Cryo-EM specimen preparation and data collection

The HN complex for structural analysis was prepared by adding 1 mM AMPPNP to 4 mg/mL to the purified protein, in a buffer containing 50 mM Tris-HCl, pH 7.0, 100 mM NaCl, and 2.5 mM MnCl₂, and incubating the solution at 60°C for 5 min. To prepare HN in complex with AMPPNP and dsDNA, 1 mM AMPPNP and 12 μM dsDNA (Sub8) were added to 4 mg/mL HN complex, and the mixture was incubated at 60°C for 5 min. For the sample containing ATPγS and loop-structured DNA, 4 mg/mL HN^D49A^ complex was incubated with 1 mM ATPγS and 12 μM LoopDNA (Sub9) at 60°C for 5 min. Each prepared sample (3 μL) was supplemented with 0.001% lauryl maltose neopentyl glycol and applied to Cu 300 mesh R1.2/1.3 grids (Quantfoil) that had been glow-discharged at 10 mA for 10 s under 7 Pa, using a JEC-3000FC Auto Fine Coater (JEOL). Excess sample was blotted with Whatman #595 filter paper at a blot force of 15 for 3 s at 8°C under 100% humidity, and the grids were then plunge frozen in liquid ethane cooled to liquid nitrogen temperature, using a Vitrobot Mark IV (Thermo Fisher Scientific). Cryo-EM data were acquired using a CRYO ARM 300 (JEOL) equipped with a cold field emission gun, an in-column omega energy filter, and a GATAN K3 camera (EM01CT at SPring-8). Movies were recorded in the CDS electron counting mode at a nominal magnification of 60,000×, with a pixel size of 0.752 Å or 0.8 Å, a total electron dose of ~50 e^−^/Å², and 50 frames per stack. Automated data collection was carried out using the SerialEM software (53), with a defocus range from −1.0 to −3.8 µm (target defocus range after stage shift: −1.2 to −1.4 µm), using a Multi-Shot pattern of 5 × 5 × 1 (or ×2) with active beam tilt compensation.

### Image processing

Data processing was performed using the CryoSPARC v4.3.1 software package (27). Beam-induced motion correction was applied to the movie stacks, and contrast transfer function (CTF) parameters were estimated. Initial particle picking was carried out using the blob picker on 50 micrographs, followed by particle selection using Topaz (54) or template-based picking. Particles were extracted under the following conditions: AMPPNP-bound form without dsDNA (box size 500 pixels and Fourier crop 120 pixels), dsDNA-bound AMPPNP form (box size 520 pixels and Fourier crop 260 pixels), and dsDNA-bound ATPγS form (box size 400 pixels and Fourier crop 100 pixels). Extracted particles were subjected to 2D classification, ab initio reconstruction, and heterogeneous refinement. Prior to refinement, particles were re-extracted at box sizes of 500, 520, and 400 pixels, respectively, without cropping. Subsequently, the refined particles underwent reference-based motion correction and were further classified through 3D classification. As a result, representative classes were selected for both the HN complex with and without DNA and then subjected to non-uniform refinement and CTF refinement. This process yielded cryo-EM maps at overall resolutions of 2.81 Å (AMPPNP form without dsDNA, 204,172 particles), 2.3 Å (dsDNA-bound AMPPNP form, 256,392 particles), 3.09 Å (ATPγS form state 1, 37,584 particles), 2.97 Å (ATPγS form state 2, 60,765 particles), and 3.14 Å (ATPγS form state 3, 96,354 particles). The resolution of each map was determined based on the gold-standard Fourier shell correlation criterion of 0.143. Angular distribution was estimated using CryoSPARC. Statistical information related to the cryo-EM data processing is summarized in Table S1.

### Model building

The initial NurA and HerA protein models were generated using AlphaFold2 (55). These models were fitted into the cryo-EM map of the AMPPNP-bound form without dsDNA, using the fit-in-map tool in UCSF ChimeraX (56) and UCSF Chimera (57). The atomic models were iteratively rebuilt manually and refined using Coot (58) and Phenix (59, 60), respectively. For the other four models, the refined model served as the template and was subjected to rigid-body rotation, followed by additional manual rebuilding with Coot and refinement using Phenix. Sharpened maps were used only for model-building, whereas the final refinement and validation were performed against the unsharpened maps. Statistics for the refined models are summarized in Table S2. All structural figures were generated using ChimeraX.

## Data Availability

The atomic coordinates of the HN complex of the AMPPNP form without dsDNA, the AMPPNP form with dsDNA, and the ATPγS form with dsDNA in states 1, 2, and 3 were deposited in the Protein Data Bank under accession codes 9X1L, 9X1M, 9X1N, 9X1O, and 9X1P, respectively. The corresponding cryo-EM maps are available in the Electron Microscopy Data Bank under accession codes EMD-66462, EMD-66463, EMD-66464, EMD-66465, and EMD-66466, respectively.

## SUPPLEMENTAL MATERIAL

Supplemental Material mbio.03523-25-s0001.pdf Tables S1 to S3 and Fig. S1 to S14.

Movie S1 mbio.03523-25-s0002.mp4 Structural flexibility of the HN complex.

ASM does not own the copyrights to Supplemental Material that may be linked to, or accessed through, an article. The authors have granted ASM a non-exclusive, world-wide license to publish the Supplemental Material files. Please contact the corresponding author directly for reuse.
