# Supplementary material for: Substrate specificity and action mechanism of the HerA-NurA nuclease from the hyperthermophilic archaeon Thermococcus kodakarensis
Source: mBio. 2026 Feb 5;17(3):e03523-25. doi: 10.1128/mbio.03523-25 (PMC12977600; doi:10.1128/mbio.03523-25)
Supplement: Supplemental Material — Tables S1 to S3 and Fig. S1 to S14. [file mbio.03523-25-s0001.pdf]

Supplementary Information

Substrate specificity and action mechanism of the HerA-NurA nuclease from the hyperthermophilic archaeon *Thermococcus kodakarensis*

Keishiro Uda[1], Takeshi Yamagami[1], Sonoko Ishino[1,4], Christoph Gerle[2], Chai C. Gopalasingam[2], Hideki Shigematsu[3], Tomoyuki Numata[1]*, and Yoshizumi Ishino[1,4,5]*

[1] Department of Bioscience and Biotechnology, Graduate School of Bioresource and Bioenvironmental Sciences, Kyushu University, Fukuoka 819-0395, Japan

[2] Life Science Research Infrastructure Group, RIKEN SPring-8 Center, Hyogo 679-5148, Japan

[3] Diffraction and Scattering Division, Japan Synchrotron Radiation Research Institute, SPring-8, Hyogo 679-5198, Japan

[4] Nagahama Institute of Bio-Science and Technology, Shiga 526-0829, Japan

[5] Institute of Innovative Research, Institute of Science Tokyo, Kanagawa 226-8503, Japan

* Correspondence should be addressed to

ishino@agr.kyushu-u.ac.jp or tomoyuki.numata94@agr.kyushu-u.ac.jp

744 Motooka, Nishi-ku, Fukuoka-shi, Fukuoka 819-0395, Japan

Table S1 Cryo-EM data collection variability.

	HN-AMPPNP complex	HN-AMPPNP-dsDNA complex	HN-ATPγS-dsDNA complex		
			State 1	State2	State3
EMDB ID	EMD-66462	EMD-66463	EMD-66464	EMD-66465	EMD-66466
Microscope	CRYOARM™ 300				
Magnification	60,000				
Voltage (kV)	300				
Detector	Gatan K3 camera				
Automation software	SerialEM				
Electron exposure (e^-/ Å^2)	50				
Defocus range (μm)	-1.4 to -1.6				
Pixel size (Å)	0.8	0.752			
Symmetry imposed	C_2	C_1	C_1		
Movies (no.)	4,750	5,600	10,350		
Initial particles images (no.)	869,353	1,425,408	4,824,374		
Final particles images (no.)	204,172	256,392	37,584	60,765	96,354
Map resolution (Å)	2.81	2.3	3.09	2.97	3.14
FSC threshold	0.143				
Map sharpening B factor (Å^2)	-105.1	-57.8	-69.0	-73.1	-76.1

Table S2 Refinement statistics.

	HN-AMPPNP complex	HN-AMPPNP-dsDNA complex		HN-ATPγS-dsDNA complex	
PDB ID	9X1L	9X1M	9X1N	9X1O	9X1P
Refinement Progress			Phenix/Coot/Chimera/ChimeraX		
Initial model used	AlphaFold2	HN-AMPPNP compex		HN-AMPPNP-dsDNA compelx	
Model resolution (Å)	3.1	2.6	3.5	3.4	3.7
FSC threshold			0.5		
Model-map CC (CC mask/CC box)	0.86/0.88	0.86/0.83	0.82/0.85	0.85/0.85	0.74/0.78
Model composition					
Non-hydrogen atoms	33946	34604	34582	34977	34280
Protein residues	4278	4260	4258	4258	4258
Nucleotide residues	0	40	40	60	26
Ligands	ANP: 6	MN: 8, ANP: 6	MN: 8, AGS: 6	MN: 5, AGS: 3, ADP: 3	MN: 5, AGS: 3, ADP:3
Average B factors (Å^2)					
Protein	53.34	81.28	96.26	117.33	134.29
Nucleotide	N/A	73.36	71.02	246.8	204.2
Ligand	56,18	84.42	22.67	105.99	59.64
R.M.S. deviations					
Bonds length (Å)	0.003	0.004	0.002	0.002	0.003
Bond angles (°)	0.461	0.473	0.421	0.439	0.525
Validation					
MolProbity score	1.31	1.53	1.37	1.38	2.28
Cβ outliers (%)	0	0	0	0	0
CaBLAM outliers (%)	2.86	1.82	1.97	1.94	2.82
Clash score	2.8	6.52	5	5.14	9.42
Rotamer outliers (%)	1.22	0.59	0.89	0.53	4.39
Ramachandran plot					
Favored (%)	96.99	97	97.45	97.45	95.73
Allowed (%)	3.01	3	2.55	2.55	4.27
Disfavored (%)	0	0	0	0	0

Table S3 Oligonucleotides used for cloning and mutagenesis.

Name	Sequence (5′–3′)
nurA-F	CGCGCATATGTACAGGCTCATCGACAGGAGG
nurA-R	CCCGCGGCCGCTCACTCCAGCGGACTTCTTCCGTAC
herA-F	CGCGCATATGAGGATAGCCGAGGATATTAAC
herA-R	CCCGCGGCCGCTCAAAAGTCAACCTCGATGCCC
NurA-D49A-F	TCTACGCCGTTGCCGGAAGCC
NurA-D49A-R	GGCTTCCGGCAACGGCGTAGA
NurA-K407A-F	GCACACGAGGGCGTCGCGATCGAAAAGAAGGC
NurA-K407A-R	GCCTTCTTTTCGATCGCGACGCCCTCGTGTGC
NurA-E409A-F	GGGCGTCAAGATCGCAAAGAAGGCCTTTGAG
NurA-E409A-R	CTCAAAGGCCTTCTTTGCGATCTTGACGCCC
NurA-K410A-F	GCGTCAAGATCGAAGCGAAGGCCTTT
NurA-K410A-R	AAAGGCCTTCGCTTCGATCTTGACGC
NurA-K411A-F	GTCAAGATCGAAAAGGCGGCCTTTGAGGCC
NurA-K411A-R	GGCCTCAAAGGCCGCCTTTTCGATCTTGAC
NurA-E414A-F	GAAGGCCTTTGCGGCCGAACTT
NurA-E414A-R	AAGTTCGGCCGCAAAGGCCTTC
HerA-Q262A-F	GCAACGCGAGCATAGCGCGCTCCTACCTGC
HerA-Q262A-R	GCAGGTAGGAGCGCGCTATGCTCGCGTTGC
HerA-T325A-F	CGGAGAAGGAGGCGATAATGAGGCTTAC
HerA-T325A-R	GTAAGCCTCATTATCGCCTCCTTCTCCG
HerA-R328A-F	GAAGGAGACGATAATGGCGCTTACCATGAAAGTC
HerA-R328A-R	GACTTTCATGGTAAGCGCCATTATCGTCTCCTTC
HerA-K332A-F	CGATAATGAGGCTTACCATGGCAGTCTCCCGCTTCCTG
HerA-K332A-R	CAGGAAGCGGGAGACTGCCATGGTAAGCCTCATTATCG
HerA-R466A-F	GCAAGGGAGGGAGCGAAGTTCGGCGTCG
HerA-R466A-R	CGACGCCGAACTTCGCTCCCTCCCTTGC
HerA-K467A-F	GGGAGGGAAGGGCGTTCGGCGTCGG
HerA-K467A-R	CCGACGCCGAACGCCCTTCCCTCCC

A

Number	Name	Label	Length (nt)	Sequence (5′–3′)
1	70-Cy5	3′ Cyanine5 (Cy5)	70	GCAATTCGATCGTTGACATCTCGCGTGCTCGGTCAATCGGCAGATGCGGAGT-GAAGTTCCAACGTT*C*G*G*C
2	FAM-70	5′ FAM	70	G*C*C*G*AACGTTGGAACTTCACTCCGCATCTGCCGATTGACCGAGCACGCG-AGATGTCAACGATCGAATTGC
3	50-Cy5	3′ Cy5	50	TCGCGTGCTCGGTCAATCGGCAGATGCGGAGTGAAGTTCCAACGTT*C*G*G*C
4	FAM-50	5′ FAM	50	G*C*C*G*AACGTTGGAACTTCACTCCGCATCTGCCGAT TGACCGAGCACGCGA
5	30-Cy5	3′ Cy5	30	CAGATGCGGAGTGAAGTTCCAACGTT*C*G*G*C
6	FAM-30	5′ FAM	30	G*C*C*G*AACGTTGGAACTTCACTCCGCATCTG
7	70-F	none	70	GAGTTCGTGTCCGTACAACTGGCGTAATCATGGCCCTTCGGGGCCATTGTTT-CTCTGTGGAGGAGTCCAT
8	70-R	none	70	ATGGACTCCTCCACAGAGAAACAATGGCCCCGAAGGGCCATGATTACGCCAG-TTGTACGGACACGAACTC
9	LoopDNA	none	98	AGTAGAAAGCCAATCCACCAAAAAGACCCTGAACGAGAGCCTGGACCCCCCC-GTCCAGGCTCTCGTTCAGGGTCTTTTTGGTGGATTGGCTTTCTACT
10	Cy5-hel1	5′ Cy5	54	TCACTCCGCATCTGCCGATTCTGGCTGTGGCGTGTTTCTGGTGGTTCCTAGGTC
11	hel2	none	70	GACCTAGGAACCACCAGAAACACGCCACAGCCAGGAAGCCGATTGCGAGGC-CGTCCTACCATCCTGCAGG
12	trap	none	34	GACCTAGGAACCACCAGAAACACGCCACAGCCAG

B

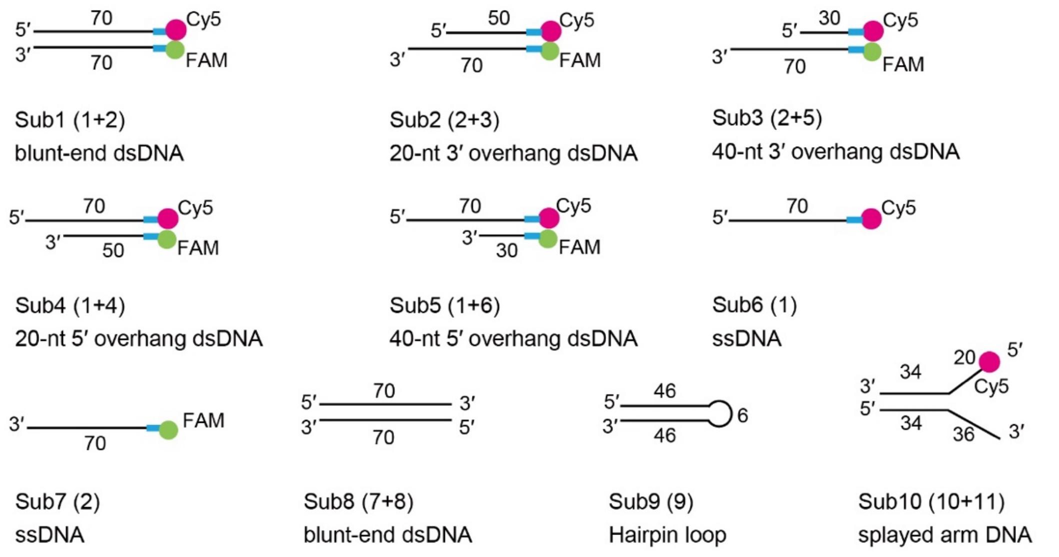

Fig S1 Substrates used in this study. (A) List of oligonucleotides used to prepare substrates for the nuclease assay. Asterisks in the sequences represent phosphorothioate modifications to prevent digestion. (B) Schematic illustrations of the substrates. The magenta circle, green circle, and cyan bar represent Cy5 labeling, FAM labeling, and phosphorothioate modification, respectively. The numbers on the bars indicate the strand lengths (nt). The substrate names and combinations of the numbers of the annealed oligonucleotides on the left side of (A) are shown under each substrate DNA.

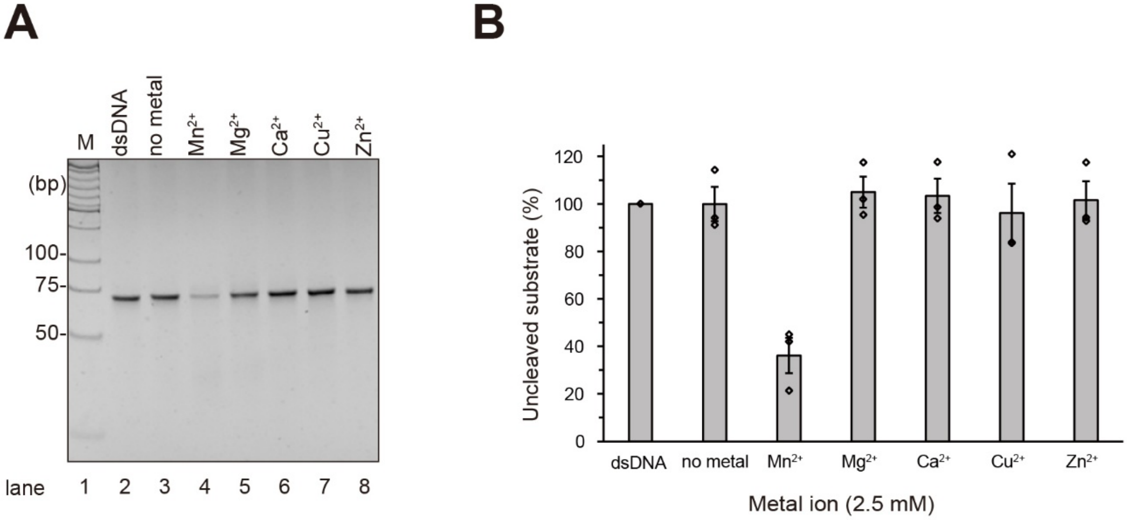

Fig S2 Metal ion dependency of the nuclease from the HN complex. (A) The metal ions used for the reactions are indicated above the gel image. M, DNA size markers; dsDNA, substrate (Sub8) as a control. (B) Quantitative analyses of the band intensities in (A). The error bars represent the standard error of the mean (SEM) from three independent experiments.

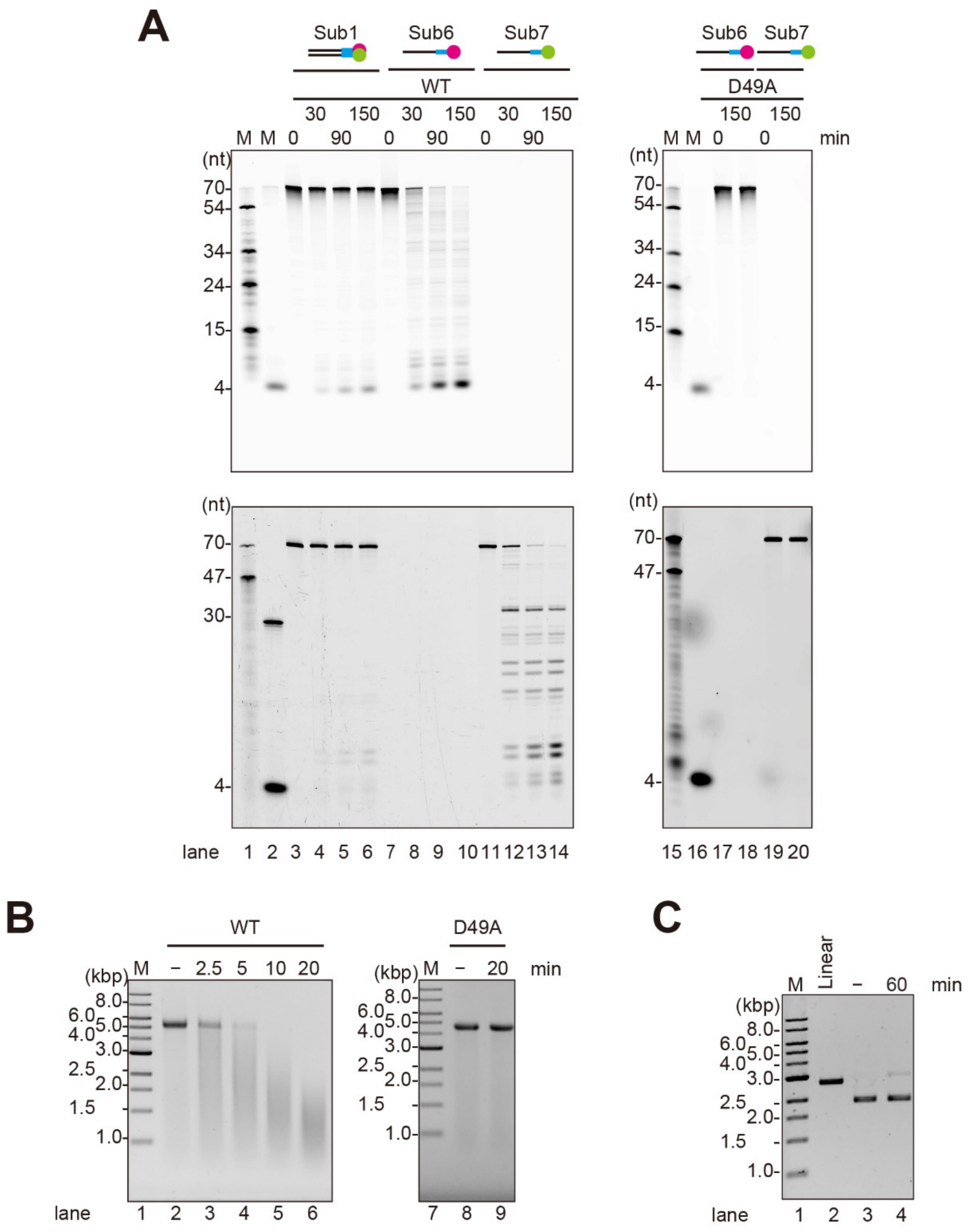

Fig S3 Nuclease activity of NurA. (A) The linear substrates are schematically illustrated above the panel. The Cy5-detected gel images (top panels) and the FAM-detected gel images (bottom panels) are shown. The reaction was performed under the same conditions as for the HN complex shown in Fig 1, with a 60-fold excess amount of NurA for the time indicated on each lane of the gel. DNA size markers were run in lanes 1 and 2 (indicated as 'M'), and their sizes (nt) are shown on the left of the panels. The parallel reactions with the NurA

protein with the D49A mutation (NurA^{D49A}) are shown on the right. (B) Endonuclease activity assay of NurA, using phiX174 virion DNA as a single-stranded circular DNA. Reaction products were separated by agarose gel electrophoresis, stained with ethidium bromide, and visualized by UV illumination. The reactions using NurA^{D49A} are shown on the right. Reaction time is indicated as a number above each lane. DNA size markers were run in lane 1 (indicated as 'M'), and their sizes (kbp) are shown on the left of the panels. (C) Endonuclease activity assay of NurA, using pUC18 DNA as a double-stranded circular DNA. The reaction and gel electrophoresis analysis were performed in the same manner as in (B). Reaction time is indicated as a number above each lane. M, DNA size markers (kbp); linear, HindIII-digested pUC18; –, incubation without NurA.

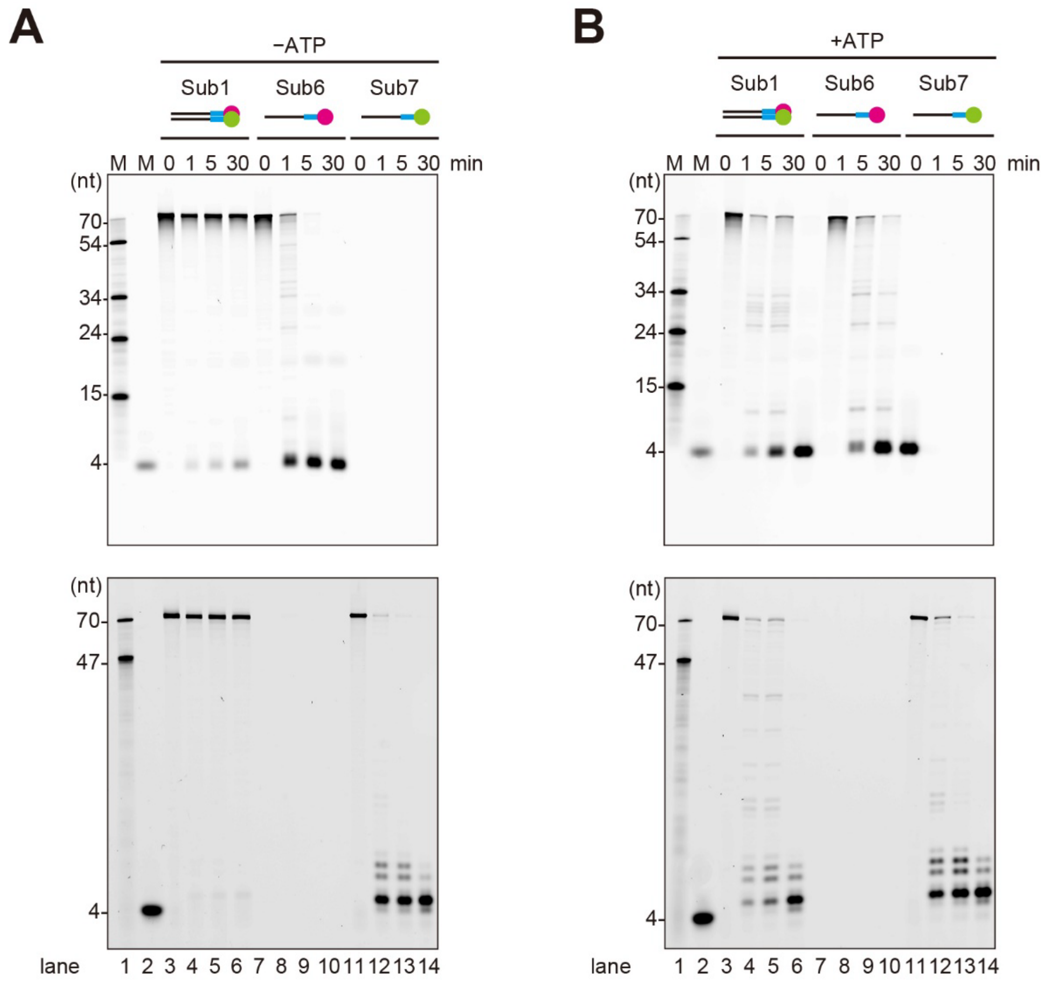

Fig S4 Nuclease activity assay of the HN complex with dsDNA and ssDNA in the absence (A) and presence (B) of ATP. The substrates are schematically illustrated above each panel. The Cy5-detected gel images (top panels) and the FAM-detected gel images (bottom panels) are shown. The reaction time is indicated as a number above each lane. DNA size markers were run in lanes 1 and 2 (indicated as 'M'), and their sizes (nt) are shown on the left of the panels.

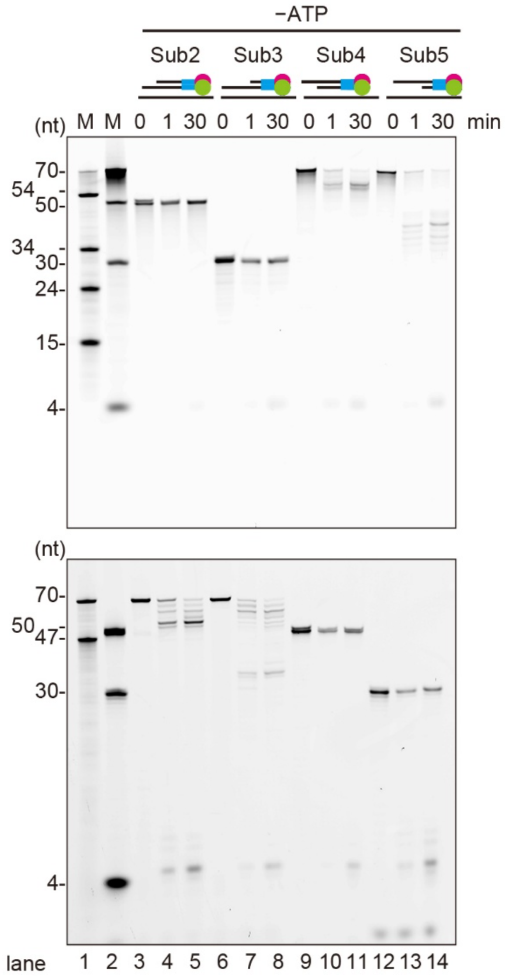

Fig S5 Nuclease activity of the HN complex in the absence of ATP. The substrates are schematically illustrated above the panel. The Cy5-detected gel images (top panel) and the FAM-detected gel images (bottom panel) are shown. The reaction time is indicated as a number above each lane. DNA size markers were run in lanes 1 and 2 (indicated as 'M'), and their sizes (nt) are shown on the left of the panels.

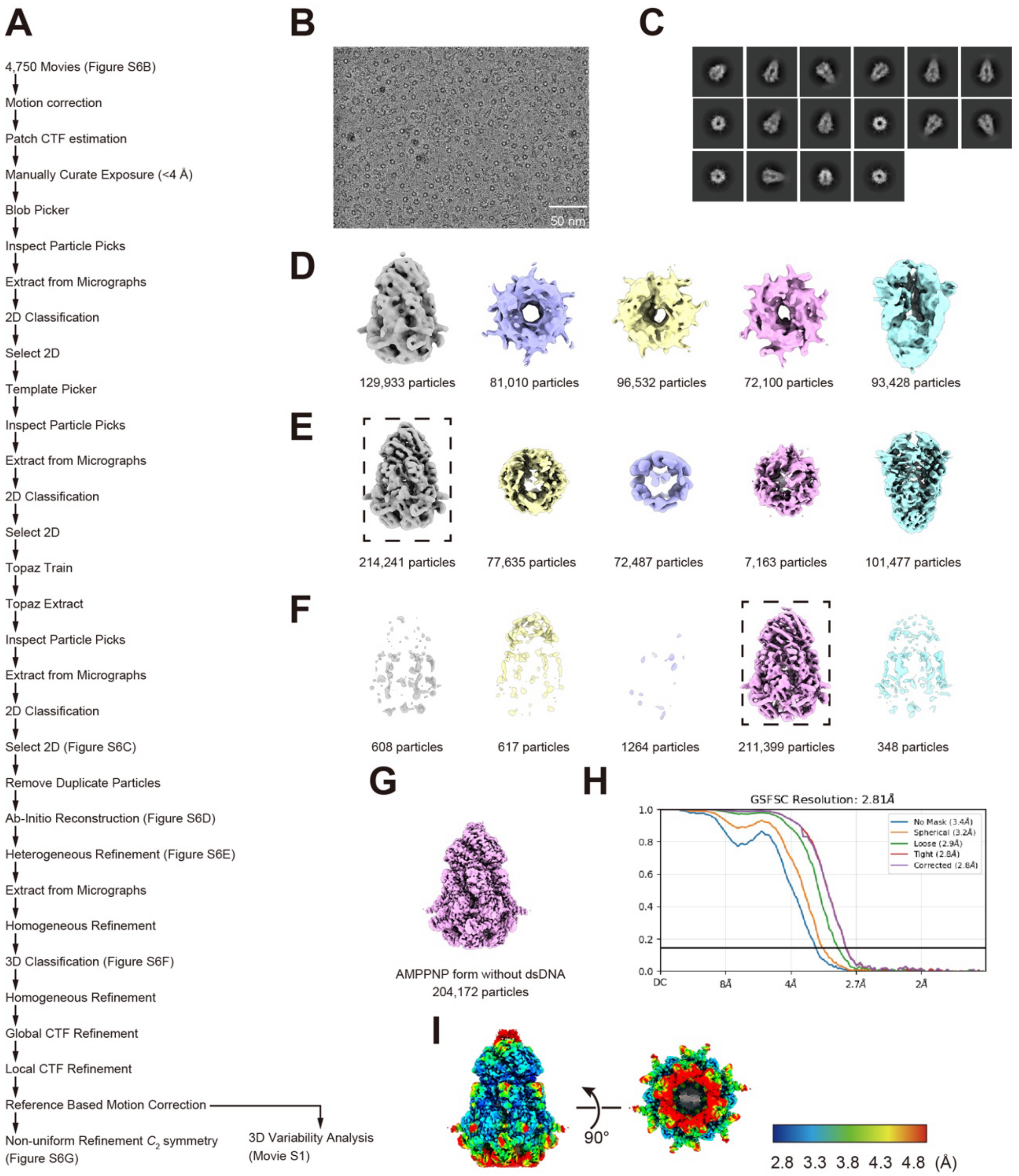

Fig S6 Cryo-EM single-particle analysis of the HN-AMPPNP complex. (A) The single-particle analysis workflow. (B) Representative motion-corrected micrograph. (C) Selected 2D class-averages for Ab-initio Reconstruction and further steps. (D) Maps from Ab-initio Reconstruction. (E) Maps from heterogeneous refinement. The map enclosed in the dotted box indicates the class selected for subsequent analysis. (F) Maps classified by 3D classification without alignment in CryoSPARC. The map enclosed in the dotted box denotes the class selected for further analysis. (G) The 3D map refined by Non-Uniform Refinement in CryoSPARC. (H) Gold-standard Fourier shell correlation (FSC) curve. The horizontal line indicates the FSC at 0.143. (I) Estimated local resolution colored on the map.

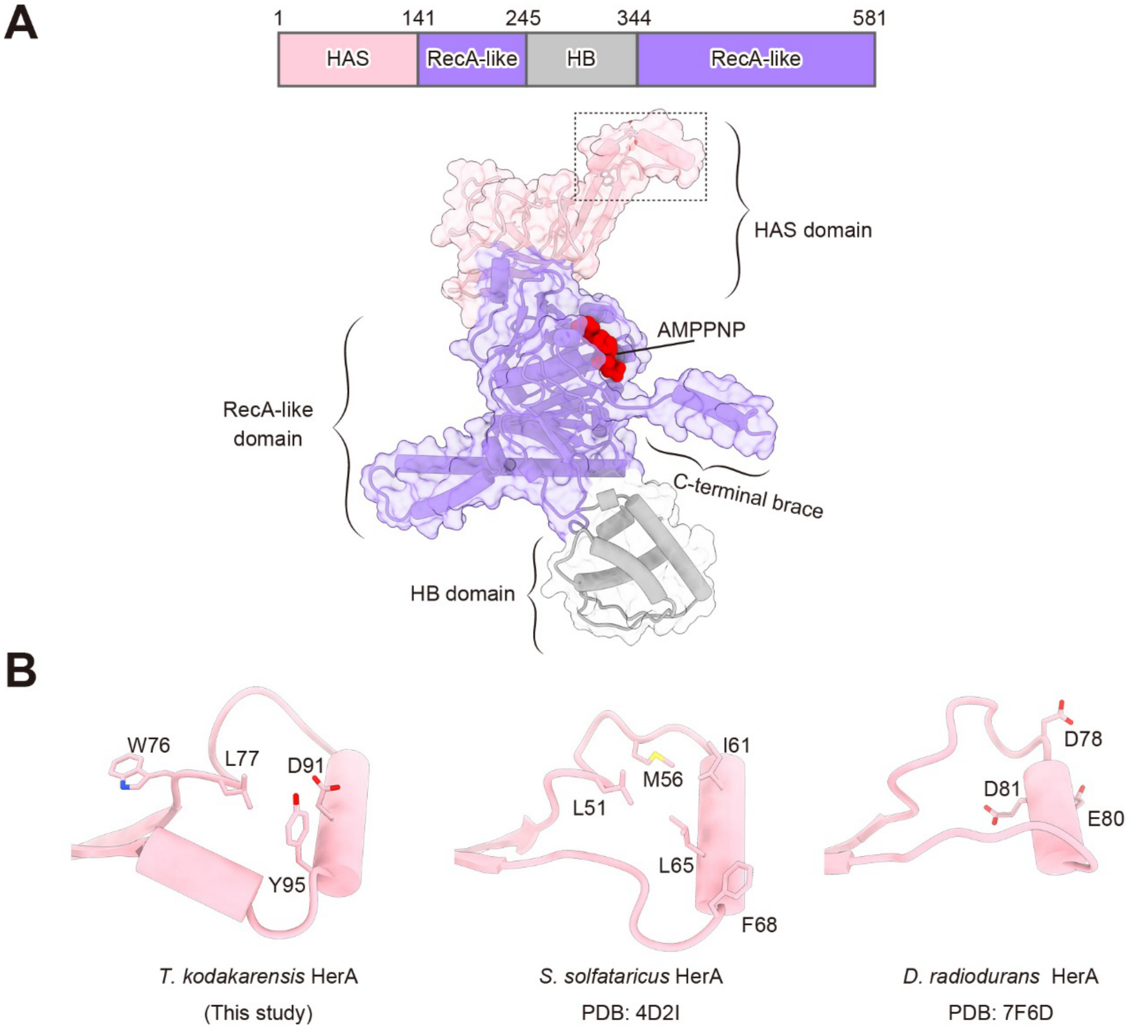

Fig S7 Structure of HerA protomer in AMPPPNP form without dsDNA. (A) Domain architecture and structure of HerA subunit from *T. kodakarensis*. The domains are color-coded as follows: HAS is pink, RecA-like is purple, and HB is light gray. The bound AMPPNP is colored red. The C-terminal brace is indicated. (B) Structural comparison of the NurA-interaction region within the HAS domain of HerA proteins from different species. The partial HAS domain structures from *T. kodakarensis* (left), *S. solfataricus* (middle), and *D. radiodurans* (right). The close-up region is indicated by the black dotted box in panel (A). Residues involved in interactions with NurA are shown as sticks.

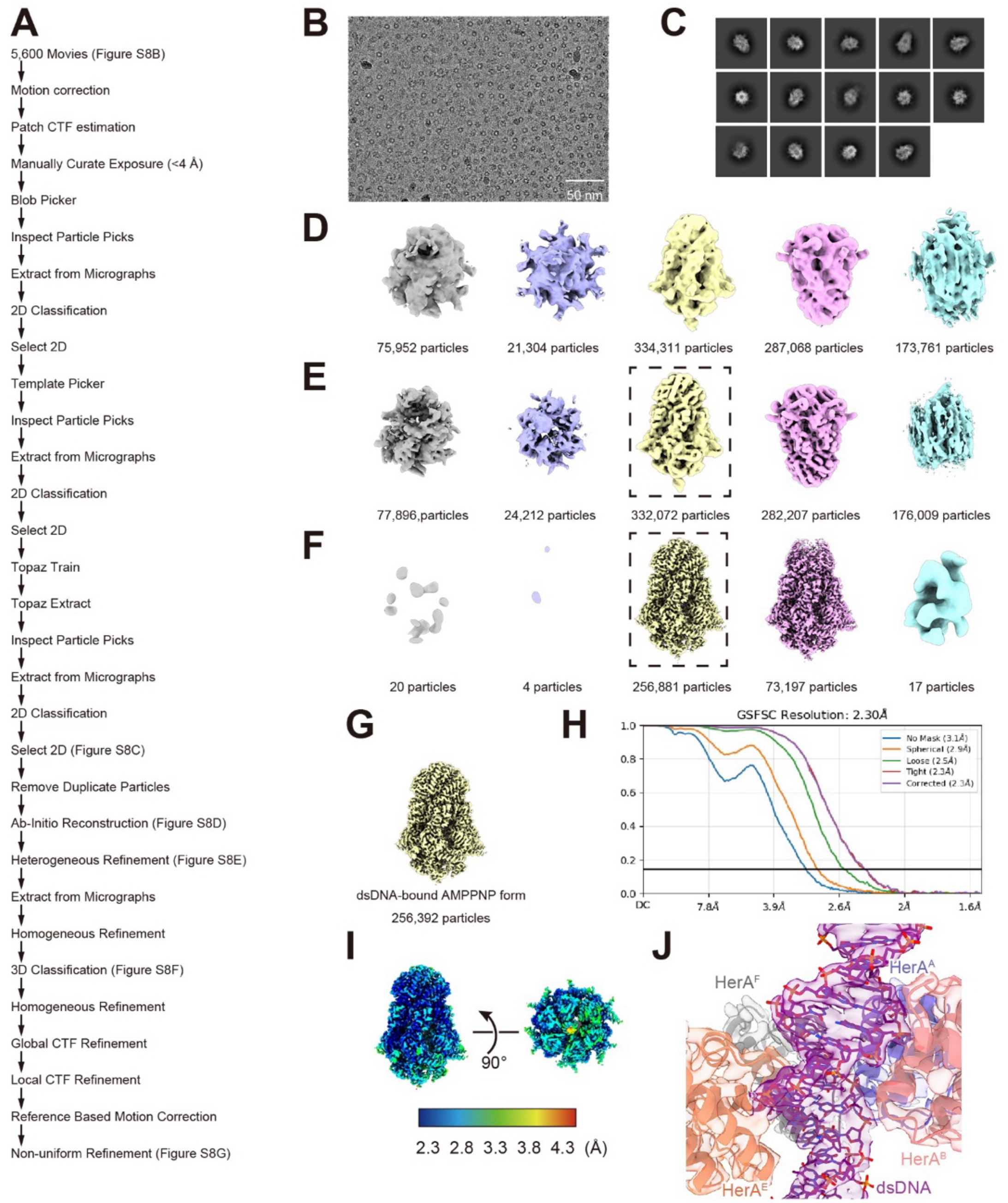

Fig S8 Cryo-EM single-particle analysis of the HN-AMPPNP-dsDNA complex. (A) The single-particle analysis workflow. (B) Representative motion-corrected micrograph. (C) Selected 2D class-averages for Ab-initio Reconstruction and further steps. (D) Maps from Ab-initio Reconstruction. (E) Maps from heterogeneous refinement. The map enclosed in the dotted box indicates the class selected for subsequent analysis. (F) Maps classified by 3D classification without alignment in CryoSPARC. The map enclosed in the dotted box denotes the class selected for further analysis. (G) The 3D map refined by Non-Uniform Refinement in CryoSPARC. (H) Gold-standard Fourier shell correlation (FSC) curve. The horizontal line indicates the FSC at 0.143. (I) Estimated local resolution colored on the map. (J) Model fitted into the map of the dsDNA and HB domain of HerA regions.

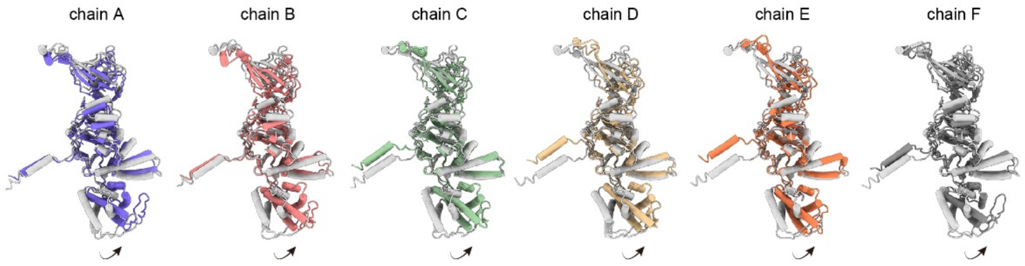

Fig S9 Structural comparison of HerA subunits with or without dsDNA. Each HerA protomer was isolated from the HN-AMPPNP complex structures with (colored) and without (light gray) dsDNA (Figs 3A and 4A) and superimposed individually. Arrows highlight the conformational changes in the HB domain upon DNA binding.

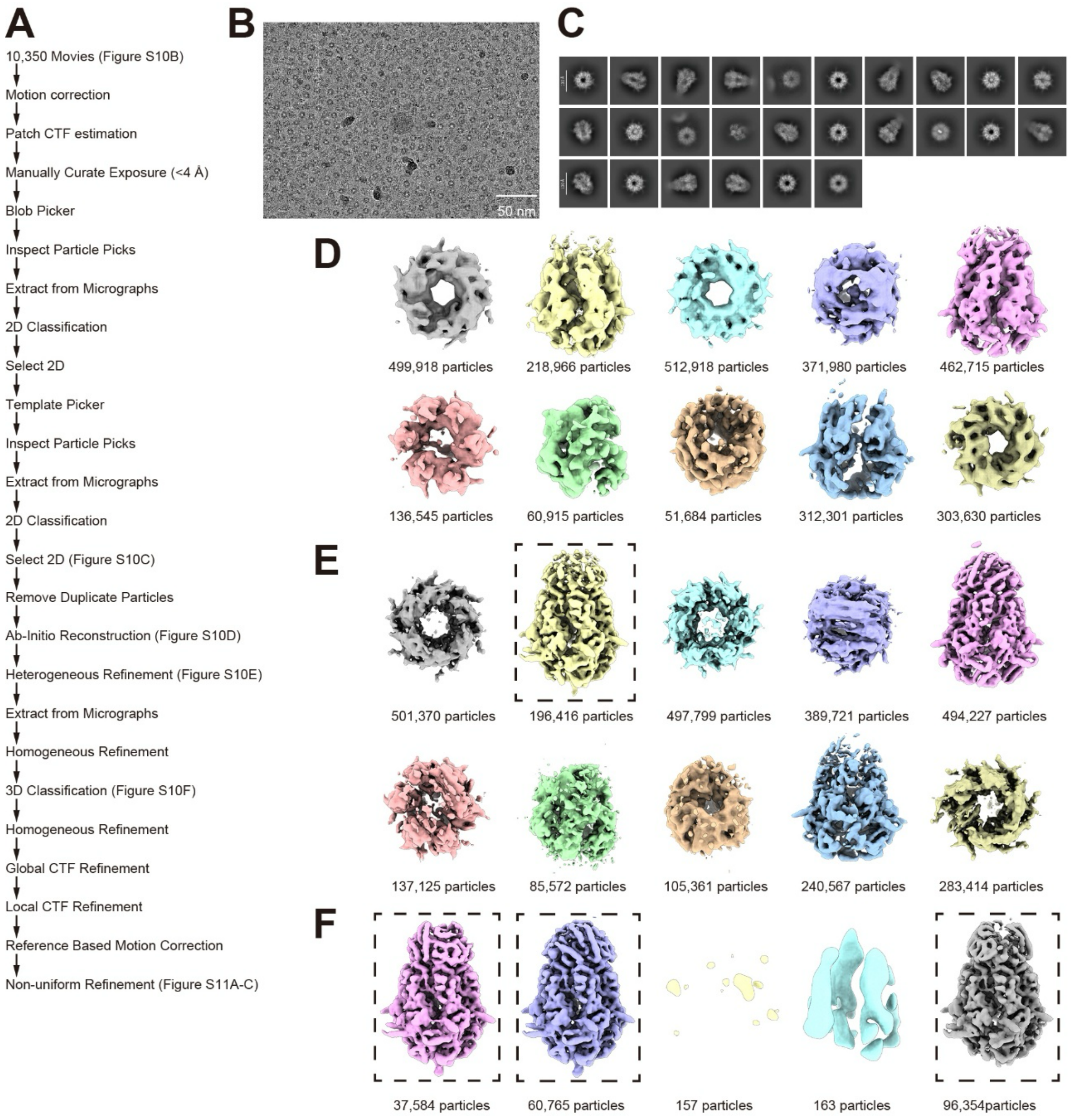

Fig S10 Cryo-EM single-particle analysis of the HN-ATPγS-dsDNA complex. (A) The single-particle analysis workflow. (B) Representative motion-corrected micrograph. (C) Selected 2D class-averages for Ab-initio Reconstruction and further steps. (D) Maps from Ab-initio Reconstruction. (E) Maps from heterogeneous refinement. The map enclosed in the dotted box indicates the class selected for subsequent analysis. (F) Maps classified by 3D classification without alignment in CryoSPARC. The map enclosed in the dotted box denotes the class selected for further analysis (see Fig S11).

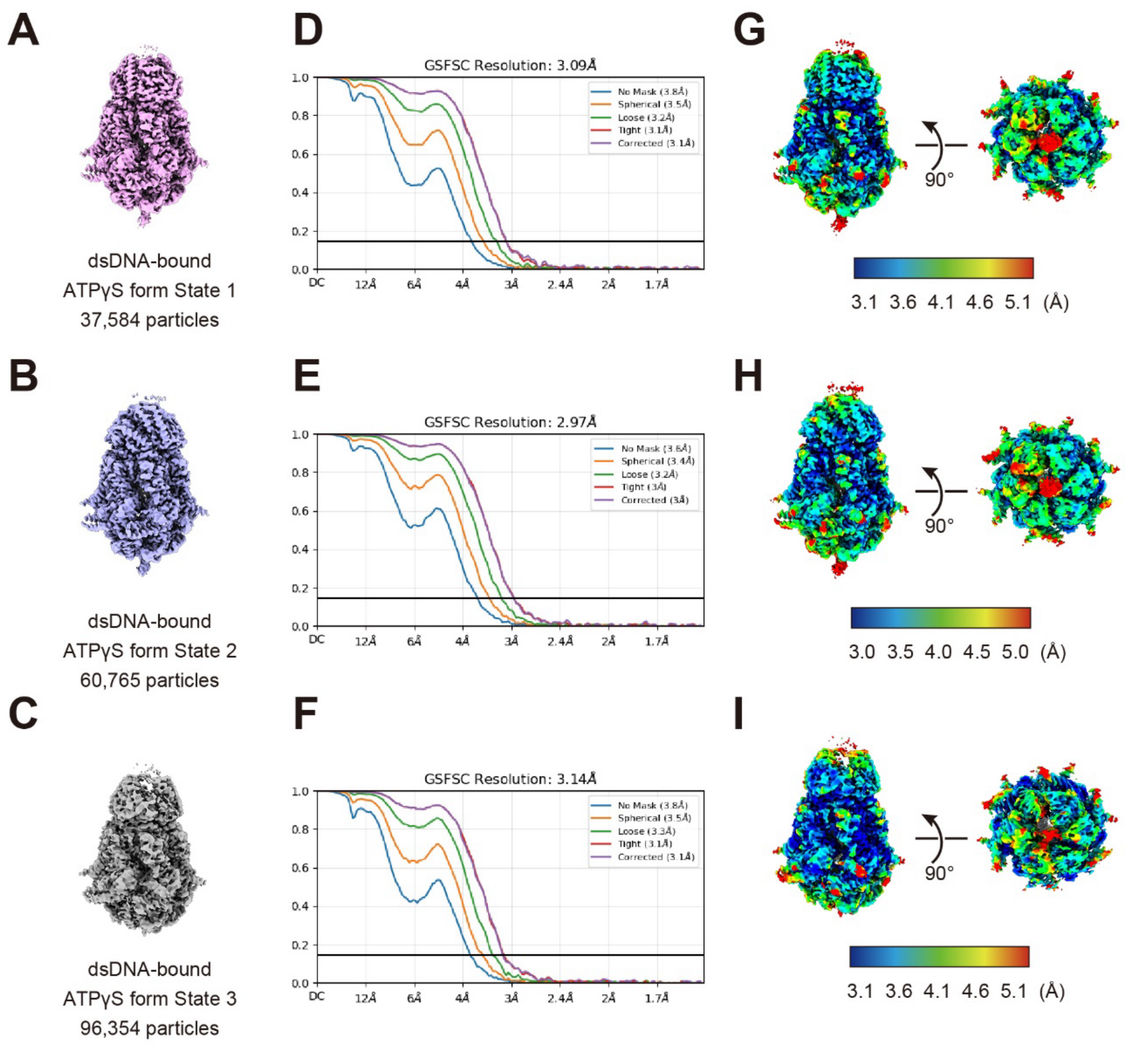

Fig S11 Refined 3D maps of the HN-ATPγS-dsDNA complex. (A-C) The 3D maps refined by Non-Uniform Refinement in CryoSPARC, as a continuation of Fig S10. (D-F) Gold-standard Fourier shell correlation (FSC) curves. The horizontal line indicates the FSC at 0.143. (G-I) Estimated local resolution colored on the map.

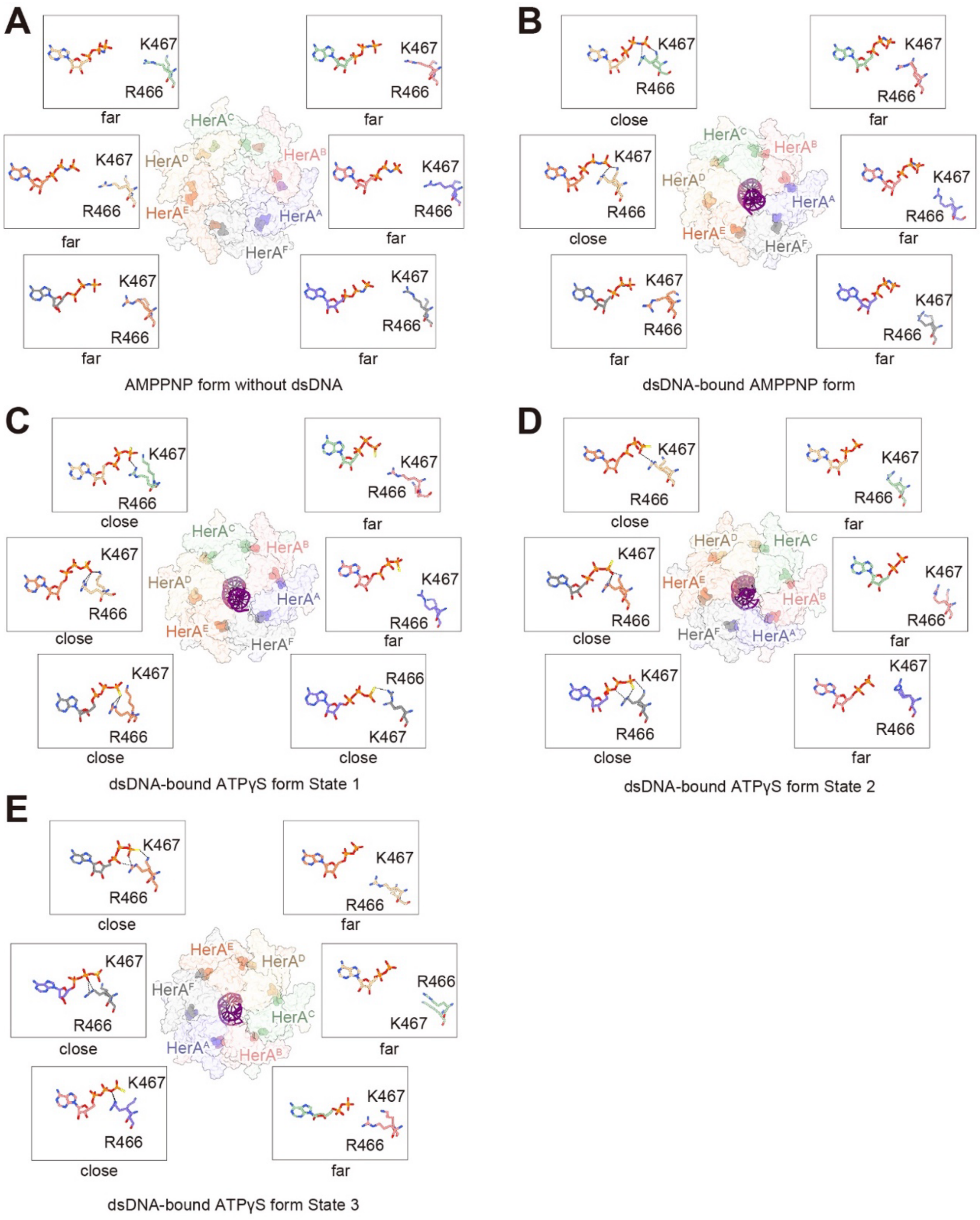

Fig S12 Structures of HerA and the bound nucleotides. The HerA structures of the HN complex viewed from the bottom. AMPPNP form without dsDNA (A), dsDNA-bound AMPPNP form (B), dsDNA-bound ATPγS form State 1 (C), State 2 (D), and State 3 (E). The color codes are consistent with those in Figs 3 and 4. The bound nucleotide and residues R466 and K467 are shown as sphere models. Each boxed panel presents a close-up view highlighting the interactions between the nucleotide and the two residues, shown as stick models. Hydrogen bonds are represented by dashed lines.

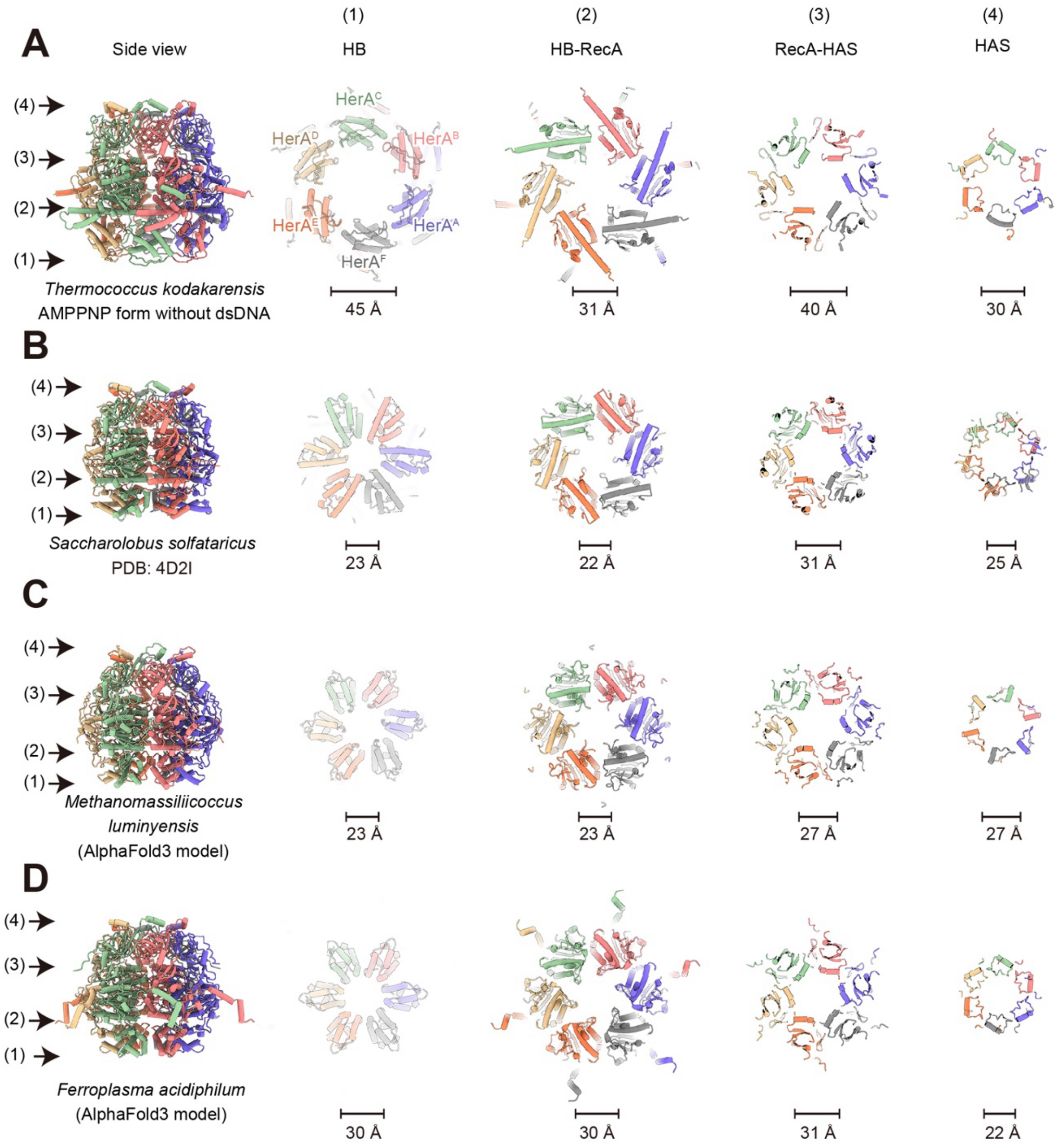

Fig S13 Comparison of HerA structures. The determined or predicted structures of thermophilic and mesophilic archaea were shown. A. *T. kodakarensis* HerA (this study); B, *S. solfataricus* HerA (PDB ID: 4D2I); C, *Methanomassiliicoccus luminyensis* HerA (Accession code: WP_019178736); D, *Ferroplasma acidiphilum* HerA (Accession code: ARD85413). A and B are determined as AMP-PNP-bound forms. C and D are predicted by AlphaFold3 (S1). A and B are derived from thermophiles. C and D are derived from mesophiles. The leftmost diagrams show a side view of each HerA hexamer. The cross-sectional views cut at four different heights are shown in the following four figures. The

cross-sections show the HB domain (1), the HB/RecA-like boundary (2), the RecA/HAS boundary (3), and the HAS domain (4) as indicated by arrows in the left side of the side views. The measured diameter of the narrowest ring is shown in each HerA ring.

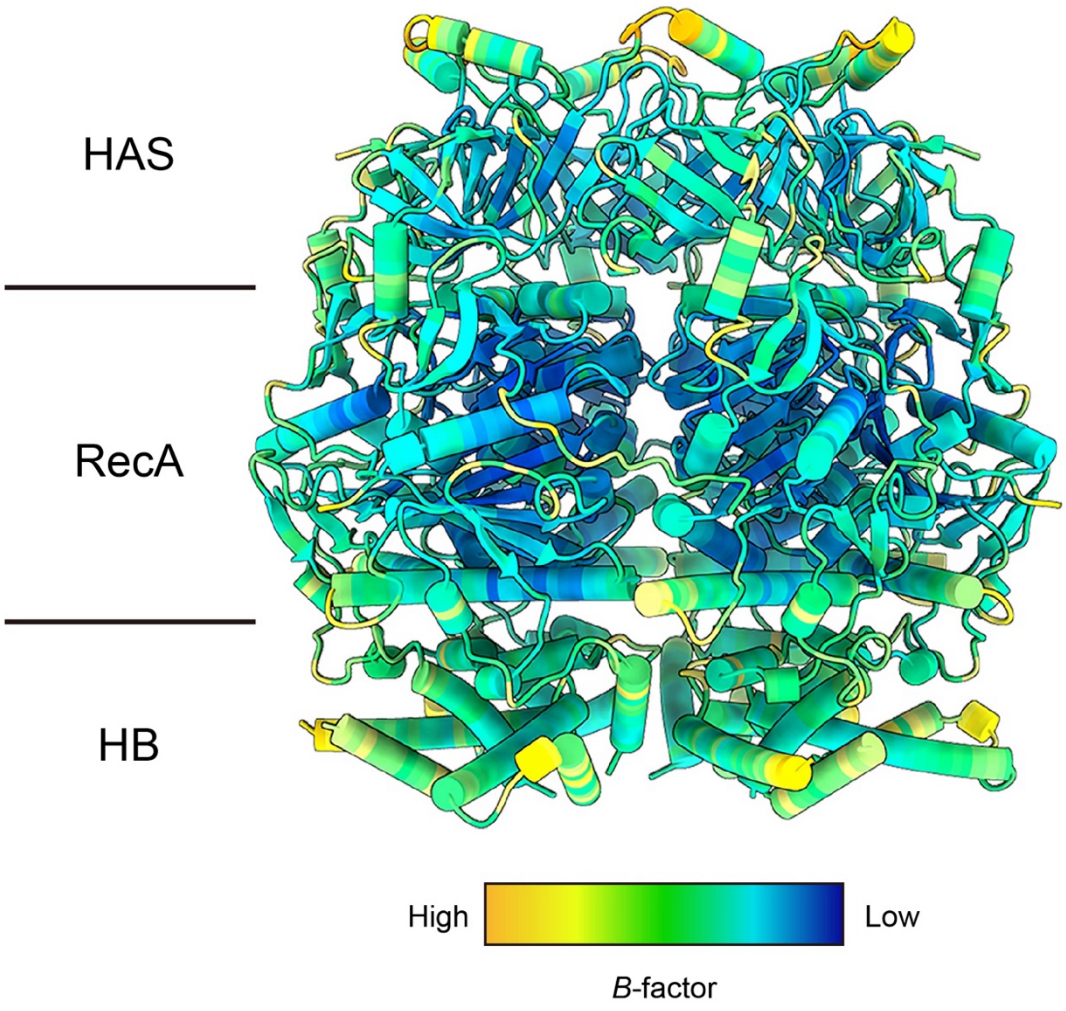

Fig S14 The HB domain is more mobile than other parts of SsoHerA. Crystal structure of SsoHerA (PDB ID: 4D2I), in which the *B*-factors of the individual regions with different colors are shown. The domains within the structure are shown on the left.

Movie S1 Structural flexibility of the HN complex. This movie visualizes the structural flexibility of the AMPPNP form without dsDNA. Each subunit is colored in the same way as in Fig 3.

Reference

S1. Abramson J, Adler J, Dunger J, Evans R, Green T, Pritzel A, Ronneberger O, Willmore L, Ballard AJ, Bambrick J, et al. 2024. Accurate structure prediction of biomolecular interactions with AlphaFold 3. Nature 630:493–500. https://doi.org/10.1038/s41586-024-07487-w
